# A tumor mutational burden-derived immune computational framework selects sensitive immunotherapy/chemotherapy for lung adenocarcinoma populations with different prognoses

**DOI:** 10.3389/fonc.2023.1104137

**Published:** 2023-06-30

**Authors:** Wenlong Zhang, Chuzhong Wei, Fengyu Huang, Wencheng Huang, Xiaoxin Xu, Xiao Zhu

**Affiliations:** ^1^Huizhou First Hospital, Guangdong Medical University, Huizhou, China; ^2^Computational Oncology Laboratory, The Marine Biomedical Research Institute, Guangdong Medical University, Zhanjiang, China

**Keywords:** lung adenocarcinoma, tumor mutational burden, prediction of prognosis, immune landscape, immunotherapy, chemotherapy, molecular docking technology

## Abstract

**Background:**

Lung adenocarcinoma (LUAD) kills millions of people every year. Recently, FDA and researchers proved the significance of high tumor mutational burden (TMB) in treating solid tumors. But no scholar has constructed a TMB-derived computing framework to select sensitive immunotherapy/chemotherapy for the LUAD population with different prognoses.

**Methods:**

The datasets were collected from TCGA, GTEx, and GEO. We constructed the TMB-derived immune lncRNA prognostic index (TILPI) computing framework based on TMB-related genes identified by weighted gene co-expression network analysis (WGCNA), oncogenes, and immune-related genes. Furthermore, we mapped the immune landscape based on eight algorithms. We explored the immunotherapy sensitivity of different prognostic populations based on immunotherapy response, tumor immune dysfunction and exclusion (TIDE), and tumor inflammation signature (TIS) model. Furthermore, the molecular docking models were constructed for sensitive drugs identified by the pRRophetic package, oncopredict package, and connectivity map (CMap).

**Results:**

The TILPI computing framework was based on the expression of TMB-derived immune lncRNA signature (TILncSig), which consisted of AC091057.1, AC112721.1, AC114763.1, AC129492.1, LINC00592, and TARID. TILPI divided all LUAD patients into two populations with different prognoses. The random grouping verification, survival analysis, 3D PCA, and ROC curve (AUC=0.74) firmly proved the reliability of TILPI. TILPI was associated with clinical characteristics, including smoking and pathological stage. Furthermore, we estimated three types of immune cells threatening the survival of patients based on multiple algorithms. They were macrophage M0, T cell CD4 Th2, and T cell CD4 memory activated. Nevertheless, five immune cells, including B cell, endothelial cell, eosinophil, mast cell, and T cell CD4 memory resting, prolonged the survival. In addition, the immunotherapy response and TIDE model proved the sensitivity of the low-TILPI population to immunotherapy. We also identified seven intersected drugs for the LUAD population with poor prognosis, which included docetaxel, gemcitabine, paclitaxel, palbociclib, pyrimethamine, thapsigargin, and vinorelbine. Their molecular docking models and best binding energy were also constructed and calculated.

**Conclusions:**

We divided all LUAD patients into two populations with different prognoses. The good prognosis population was sensitive to immunotherapy, while the people with poor prognosis benefitted from 7 drugs.

## 1 Introduction

The incidence of lung cancer is the second highest in the world (1–3). Lung cancer kills millions of people yearly, and its 5-year survival rates vary from 4-17% on the ground of stages and regional differences (4). The most common histological type of lung cancer is lung adenocarcinoma (LUAD) (5, 6). Last decade, many pioneers studied the LUAD gene, and some of these outstanding scientists achieved remarkable results. KRAS, EGFR, and BRAF are most commonly oncogenes with a mutation in LUAD. TP53, STK11, and KEAP have closely related to tumor suppressors (7).

Long non-coding RNA (lncRNA) is the over 200 bp RNA and is disabled to encode proteins. In recent studies, lncRNA was found to be associated with the development of tumors (8). There are infinite lncRNA types. And lncRNA JPX can increase the number of lung cancer cells and accelerate the growth of tumor cells (9). PD-L1 lncRNA splice isoform facilitates LUAD development by directly enhancing c-Myc activity (10). Moreover, novel lncRNA UPLA1 regulates the activity of LUAD. UPLA1 can facilitate migration, invasion, and proliferation of LUAD and is associated with cell cycle arrest (11). Thus, numerous unknown features between lncRNA and LUAD are worthy of research.

Tumor mutational burden (TMB) indicates the number of mutations per million bases. Recently, FDA approved the pembrolizumab (PD-1 antibody) for the treatment of adult and pediatric patients with unresectable or metastatic high TMB (≥10 mutations/megabase) solid tumors (https://www.accessdata.fda.gov/drugsatfda_docs/label/2020/125514s068lbl.pdf). The immune checkpoint inhibitor (ICI)-based immunotherapy has shown a strong vitality, especially ICIs targeting programmed cell death 1 (PD-1) and programmed cell death-ligand 1 (PD-L1). Yang et al. creatively proposed ICI therapy before surgical resection of the tumor, which improved the survival rate of some lung cancer patients (12). Many studies have shown that tumor patients with high TMB values can achieve better immunotherapy effects (13–16). It shows that TMB is the latest and independent signature in evaluating the efficacy of immunotherapy (17, 18). Some scholars also extended the study of TMB to lung cancer. For example, Hellmann et al. found that lung cancer patients with high TMB had a better treatment response to Nivolumab and Ipilimumab combined immunotherapy (19).

**Figure 1:**
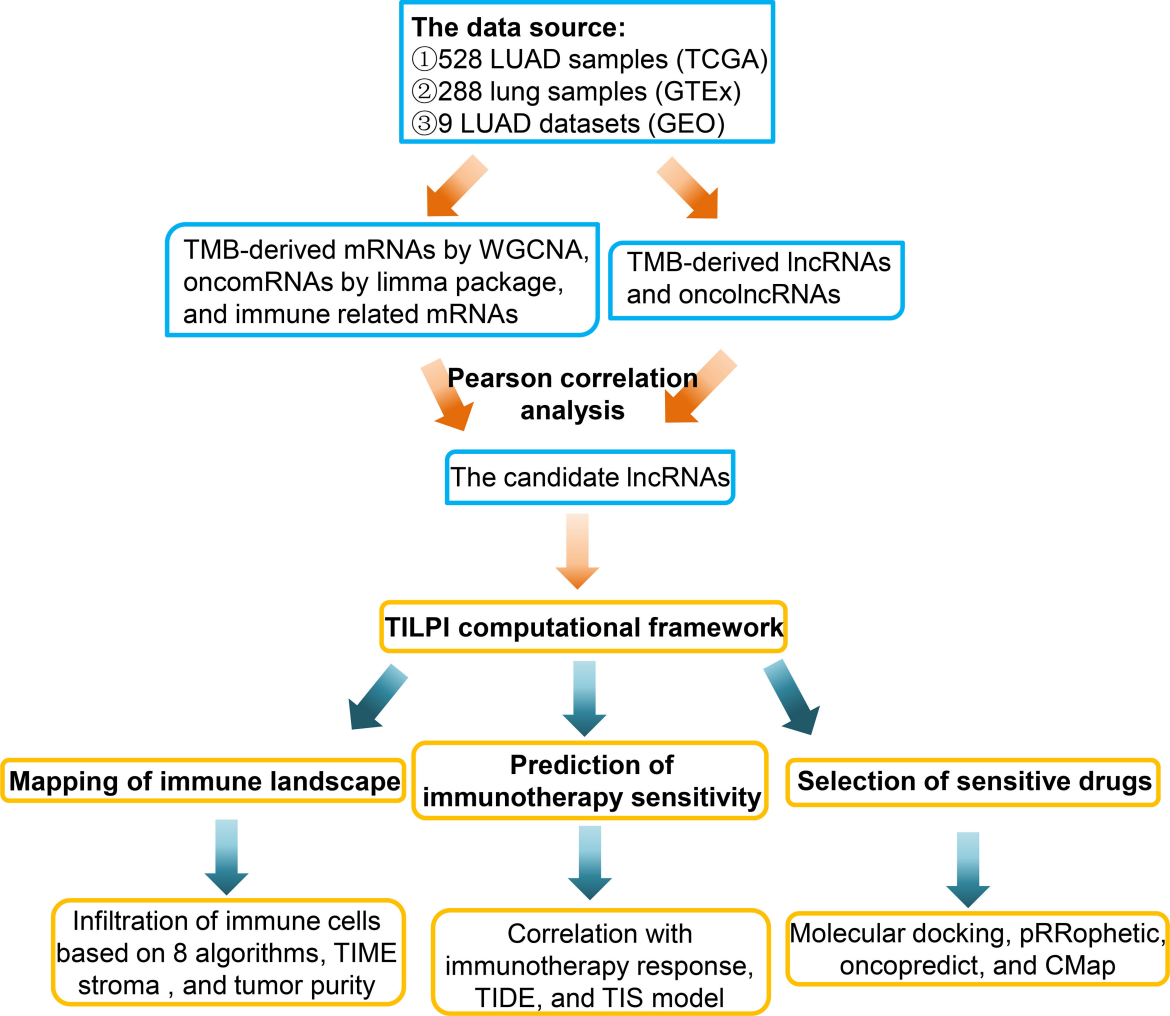
This briefly showed the experimental process of this study.

Nevertheless, now the development of TMB encountered some problems. The first was the accuracy of TMB measurement. Secondly, how to apply TMB to the prediction model was also a problem (20). The literature review found that no scholar has constructed a computing framework based on TMB to relate to patients’ prognosis and immunotherapy sensitivity. Hence, we decided to contribute in this direction (**Figure 1**).

## 2 Materials and methods

### 2.1 The datasets source

In this study, we collected 528 LUAD and 494 lung squamous cell carcinomas (LUSC) samples from The Cancer Genome Atlas (TCGA) (https://portal.gdc.cancer.gov/). The non-small cell lung cancer (NSCLC) samples consisted of aforementioned LUAD and LUSC samples. We only remained with the project of the vial with A and deleted the samples with Vial B or C. Because the vial B or C represents that corresponding samples were fixed by formalin and embedded in paraffin, the effects on RNA-sequence had been proved. We also averaged the RNA-sequence results of multiple samples from the same patient. At last, we obtained 513 LUAD samples. Furthermore, we obtained 288 normal lung samples from the Genotype-Tissue Expression (GTEx) (https://gtexportal.org/). At last, we tried to verify the reliability of the computing framework in external Gene Expression Omnibus (GEO) (https://www.ncbi.nlm.nih.gov/geo/). We collected 9 LUAD datasets with OS (1644 samples), which included GSE11969, GSE13213, GSE26939, GSE31210, GSE36471, GSE63459, GSE68465, GSE68571, and GSE72094. After sorting out these datasets, we obtained the mRNAs and lncRNAs expression matrix, overall survival (OS) time, survival status, age, gender, smoking, race, pathological stage, and pathological TNM (**Table S1**). The format of RNA-sequence data we collected was transcripts per kilobase of exon model per million mapped reads (TPM), which facilitated the validation by external datasets. The brief experimental flow was shown in the figure (**Figure 1**).

### 2.2 Identify the TMB-derived immune lncRNA set

At the same time, we calculated the TMB value of 513 LUAD samples by the TCGAmutations package of R (21). Nevertheless, we only obtained the TMB value of 509 samples. Then we listed the TMB value in a line. We picked out the 25% minimum as the low TMB group (n = 127) and the 25% maximum as the high TMB group (n = 127). These two groups were used to identify the TMB-derived genes by weighted gene co-expression network analysis (WGCNA) (22). WGCNA holds the idea that the disorder of functional networks leads the tumors and the identification of function-related genes based on the biological network would be more logical. Therefore, it researched the gene functional network analysis in multiple samples rather than simply expressed correlation. We first explored co-expression networks of genes between different TMB groups based on the WGCNA. We constructed the Topological Overlap Matrix (TOM) to decrease the noisy and false relation. Then TOM divides all genes into various module eigengenes (MEs) that consisted of similar functional genes. Then we selected the best soft powers β to build a scale-free network based on the function pickSoftThreshold. In addition, each adjacency matrix was built according to the following formula:


$$\alpha_{ij}=|S_{ij}{|}^{\beta }$$

(*α_i_*: adjacency matrix between gene i and gene j, *S_ij_*: similarity matrix done by Pearson correlation of all gene pairs, β: soft power value). And each adjacency matrix was transformed into a TOM and corresponding dissimilarity (1-TOM). Furthermore, the hierarchical clustering dendrogram based on 1-TOM was constructed, which clustered genes with similar expressions into a co-expression ME. At last, we chose MEs with high correlation coefficients (cor > 0.4, P< 0.05) to conduct further analysis. The TMB-derived lncRNAs and mRNAs of MEs with high correlation coefficients were the candidate genes to construct the computing framework. In addition, the correlation between module membership (MM) and gene significance (GS) of each ME was explored.

Not only that, we built lncRNA and mRNA differentially expression maps between 288 lung samples and 513 LUAD samples based on the limma package of R (23). There were two qualifications to screen the qualified lncRNAs and mRNAs: (1)|*log*_2_FC|≥1, FC refers to the fold change (the expression ratio of lncRNAs or mRNAs between normal samples and LUAD samples). Since the limma package just receives the expression matrix that is log bottomed by 2, so |*log*_2_FC|=1 refers to tumors expressing twice or half of lncRNAs or mRNAs than normal tissues. (2) False discovery rate (FDR)-adjusted P value< 0.05. This was aimed to obtain oncogenic lncRNAs and mRNAs.

In addition, we collected 2524 immune-related mRNAs from the immunology database and analysis portal (ImmPort) (https://www.immport.org/) and systems biology of the innate immune response (InnateDB) (http://www.innatedb.com/). Furthermore, we intersected TMB-derived mRNAs, oncogenic mRNAs, and immune-related mRNAs to determine candidate mRNAs. We also intersected TMB-derived lncRNAs and oncogenic lncRNAs to determine candidate lncRNAs. Then the Pearson analysis was conducted between candidate lncRNAs and candidate mRNAs. The high correlation coefficient and statistical meaning were qualifications of Pearson analysis (cor > 0.4, P< 0.05). In the end, we successfully identified the TMB-derived immune lncRNA set (TILncSet) and TMB-derived immune mRNA set (TImSet). The TMB-derived immune gene set (TIgeneSet) consisted of TILncSet and TImSet.

### 2.3 Explore the biological functional pathways of TIgeneSet

Curious about the biological functional pathways of TIgeneSet, we used metascape to explore the potential functions of TIgeneSet (https://metascape.org/). Firstly, we used the Molecular Complex Detection (MCODE) algorithm to construct a protein-protein interaction (PPI) network that showed the functions of TIgeneSet. Secondly, the enrichment analysis in cell type signature also identified cell types close related to TIgeneSet. Thirdly, the enrichment analysis in transcriptional regulatory relationships unraveled by sentence-based text mining (TRRUST) found potential transcription factors related to TIgeneSet. At last, the enrichment analysis in Transcription Factor Targets showed the connected targets of TIgeneSet.

Furthermore, we constructed a node network using the clusterProfiler package of R (24). We mainly used the Gene Ontology (GO) (http://geneontology.org/) and Kyoto Encyclopedia of Genes and Genomes (KEGG) (https://www.kegg.jp/) to conduct the functional enrichment analysis. The filter was P value of analysis less than 0.05.

### 2.4 Construction and validation of a computing framework for the prediction of prognosis

First, we randomly divided 513 LUAD samples into the training group (n = 257) and the testing group (n = 256). And the TCGA group (n = 513) consisted of the training group and the testing group. The aforementioned TILncSet may lead to the occurrence of LUAD. So TILncSet was more likely to take part in the LUAD progression than other lncRNAs. Therefore, we conducted a statistical analysis based on TILncSet using R-version 4.1.1. And the univariate Cox proportional risk regression analysis, multivariate Cox proportional risk regression analysis, and Kaplan-Meier (KM) method were conducted to identify significant lncRNAs to predict prognosis in the training group. The univariate Cox regression analysis and multivariate Cox regression analysis estimated the TMB-derived immune lncRNA signature (TILncSig). And the formulas of the Cox proportional risk regression analysis were as follows.


$$\text{h }\left(\text{t},\text{ X}\right)=h_{0}\left(t\right)*exp\;\left(\beta_{1}*\;X_{1}+\;\beta_{2}*\;X_{2}+\ldots +\;\beta_{n}*\;X_{n}\right)$$

On the left of the formula, h (t, X) represents the risk function of the individual where X is the predictor or covariate and t is time. The right *h*_0_(*t*) is the baseline hazard rate of h (t, X) when the X is 0, and it is the quantity to be estimated from the sample data. It’s the same for all individuals, so the only difference in risk between individuals is the difference in covariates X. The *exp* (*β*_1_*X_1_+*β*_2_*X2+···+*β_n_* *X_n_) is called the partial hazard function, and it’s different for each individual. (*β*_1_*x_1_+*β*_2_*x_2_+···+*β_n_* *x_n_) is the linear combination of covariate X. *h*_0_(*t*) is the baseline risk function, which represents the risk when all covariates X are 0. It is the same for all individuals, so the difference in risk among individuals is only the difference in covariates. It’s called the partial hazard function, and it’s different for each individual. Take the logarithm of both sides of the equation and apply the mathematical transformation:


$$\text{ln}\frac{h\;\left(t,\;X\right)}{h_{0}\;\left(t\right)}=\;\beta_{1}*\;X_{1}+\;\beta_{2}*\;X_{2}+\ldots +\;\beta_{n}*\;X_{n}$$

Then we can figure out the relative risk (RR):


$$\text{RR}=\;\frac{h\;\left(t,\;X_{i}\right)}{h\;\left(t,\;X_{j}\right)}=\;\frac{h_{0}\;\left(t\right)*\exp\;(\beta^{‘}*\;X_{i})}{h_{0}\;\left(t\right)*\exp\;(\beta^{‘}*\;X_{j})}=\;\text{exp}\;[\beta^{‘}*\left(X_{i}-\;X_{j}\right)],\;i,\;j=1,\;2,\;\ldots ,\;n$$

According to the above theory, we proposed a quantitative computing framework to predict individual prognosis in the training group.


$$\text{Risk Score}\left(\text{TILncSig}\right)=exp(\text{ln}\left(h_{0}\left(t\right)\right)+\sum_{\text{i}=1}^{\text{n}}\text{β}\left(lncRNA_{\text{i}}\right)*\text{expr}\left({\text{ lncRNA}}_{\text{i}}\;\right))$$

Risk Score (TILncSig) is the prognostic index of each LUAD patient. We also called risk score (TILncSig) as TMB-derived immune lncRNA prognostic index (TILPI). The n is the number of lncRNA signatures. β(lncRNA_i_) is the coefficient of lncRNA_i_ obtained by multivariate Cox regression analysis. Expr (lncRNA_i_) is the expression level of lncRNA_i_.

In addition, we conducted the KM method to estimate the survival probability of individuals. For the nth time point t_n_ in the study, the survival probability can be calculated as:


$$\text{S}({\text{t}}_{\text{n}})=\text{S}({\text{t}}_{\text{n}-1})\;(1-\frac{{\text{d}}_{\text{n}}}{{\text{r}}_{\text{n}}})$$

S (t_n–1_) is the probability of survival at the time point t_n–1_. d_n_ refers to the number of events occurring at the time point t_n_. r_n_ is the alive people number at time point t_n_. When t_0 = _0, that S (0) = 1.

We used the median TILPI of the training group as a boundary to judge the risk of patients. This boundary was applied to verify the reliability of the computing framework in the testing group and the TCGA group. Furthermore, we confirmed the reliability of the computing framework. First of all, we used survival analyses and 3D principal component analysis (3D PCA) to verify the difference between the high-TILPI subgroup and the low-TILPI subgroup. Secondly, the performance of TILPI was also evaluated by the time-dependent receiver operating characteristic (ROC) curve. Thirdly, the independent hazard of TILPI and the clinical characteristics were also proved in the study. Furthermore, we also verified the independence of the computing framework by grouping each clinical characteristic. And the Chi-Square test was used to prove clinical characteristics’ relationship to the computing framework. In addition, we conducted gene set enrichment analysis (GSEA) to identify the pathways enriching in different risk subgroups (25). The included KEGG pathways of GSEA were 186. And P value< 0.05 was the filter. At last, we constructed a novel model called nomogram to predict individual survival probability, which consisted of TILPI and 8 types of clinical characteristics (age, gender, smoking, race, pathological stage, and pathological TNM) (26).

### 2.5 Mapping of immune landscape based on computing framework

The tumor immune microenvironment (TIME) is the basis of immunotherapy. Based on 8 quantification algorithms, we described the immune cells’ infiltration landscape in detail. The 8 quantification algorithms were Cell type Identification by Estimating Relative Subsets of RNA Transcripts (CIBERSORT) (22 types of immune cells) (27), CIBERSORT-absolute mode (CIBERSORT-ABS) (22 types) (27, 28), Estimating the Proportions of Immune and Cancer cells (EPIC) (8 types) (29), Microenvironment Cell Populations-counter (MCPCOUNTER) (10 types) (30), Quantifying Immune Contexture of Human Tumors (QUANTISEQ) (11 types) (31), Tumor Immune Estimation Resource (TIMER) (6 types) (28, 32), Tumor and Immune System Interaction Database (TISIDB) (28 types) (33), and digitally portraying the tissue cellular heterogeneity landscape (XCELL) (36 types) (34). In addition, we picked out types of immune cells more distributed in different TILPI subgroups based on intersection analyses. There were 2 conditions for intersection analyses. Firstly, the standard-compliant immune cells must be proven more distributed in a risk subgroup with at least 2 algorithms. Secondly, this result can’t contradict another algorithm.

TIME has not only all kinds of immune cells but also numerous stromal components. Therefore, we collected the TIME score and stroma score from XCELL. And the cytotoxicity score of MCPCOUNTER was also calculated. Furthermore, we also got the stromal score and tumor purity based on an algorithm called Estimation of STromal and Immune cells in MAlignant Tumour tissues using Expression data (ESTIMATE) (35). The other TIME components were also collected from the tumor immune dysfunction and exclusion (TIDE). They included interferon gamma (IFNG), T-cell-inflamed signature (Merck18 score), CD8, CD274 (PD-L1), cancer-associated fibroblast (CAF), myeloid-derived suppressor cell (MDSC), and tumor-associated macrophage M2 (TAM M2) (36). Furthermore, we planned to obtain the immune subtype of samples based on 6 types of immune subtypes, which consisted of wound healing (immune C1), IFN-γ dominant (immune C2), inflammatory (immune C3), lymphocyte depleted (immune C4), immunologically quiet (immune C5), and TGF-β dominant (immune C6) (37). The difference and correlation analyses above were based on the Wilcoxon test and Pearson correlation coefficient.

### 2.6 Prediction of immunotherapy sensitivity based on computing framework

Immune checkpoint inhibitor (ICI) was a significant immunotherapy, which decreases the expressions of immune checkpoint proteins. It was reported that the TIDE score was excellent to predict the response to immunotherapy. Therefore, we planned to combine the TIDE score with TILPI to predict immunotherapy sensitivity. We first collected the average expression levels of cytotoxic T lymphocyte (CTL) signatures (CD8A, CD8B, GZM, GZMB, PRF1) to predict the distribution of CTL. According to the average expression levels of CTL, we divided all samples into the hot-tumor subgroups with above-average CTL levels and the cold-tumor subgroups with below-average CTL levels. Every hot tumor has a T cell dysfunction score while every cold tumor has a T cell exclusion score. In hot tumor subgroups, the T cell dysfunction score was derived by systematically identifying genes that were related to CTL infiltration levels to affect patients’ OS. T cell dysfunction score of each gene was calculated as follows:


$$\text{Dysfunction }=\frac{d}{StdErr\left(d\right)}$$

Then we compared the dysfunction scores of each gene to identify key genes that affected CTL and death hazards. For each hot-tumor sample, the final T-cell dysfunction score was modeled from Cox-PH regression:


Hazard=a*CTL+b*P +d*CTL*P

In this model, CTL represents the CTL level. The P represents the expression level of the candidate gene. The coefficient d reflects the influence of interaction between CTL and candidate gene P on death hazard. In the cold-tumor subgroup, the T cell exclusion score is derived by the expression levels of 3 types of cells that restrict T cell infiltration in tumors. They are CAF, MDSC, and TAM. T cell exclusion score of the cold-tumor subgroup was acquired from TIDE (http://tide.dfci.harvard.edu/). In the end, the TIDE score is the combination of the T cell dysfunction score from the hot-tumor subgroup and the T cell exclusion score from the cold-tumor subgroup. Based on the above computation, we analyzed the correlation between the TILPI computing framework and various scores of TIDE.

Moreover, we found a novel immune prediction model called tumor inflammation signature (TIS) (38). It has proved that the TIS model retrospectively predicted the clinical benefit of anti-PD-1 treatment in clinical trials. TIS model also quantifies an adaptive immune response in TIME. TIS model is composed of 18 genes (CD3D, IDO1, CIITA, CD3E, CCL5, GZMK, CD2, HLA-DRA, CXCL13, IL2RG, NKG7, HLA-E, CXCR6, LAG3, TAGAP, CXCL10, STAT1, GZMB). We also connected TILPI with the TIS model to predict the immunotherapy response. At last, we conducted time-dependent ROC curves for the TIDE model, TIS model, and TILPI computing frameworks in 1, 3, and 5 years’ OS.

The 5 published transcriptomics signatures of the immune response were used to validate the possibility that the low TILPI group was suitable for immunotherapy. Tertiary lymphoid structures (TLS) signature is based on differentially expressed genes in tumor tissue with TLS (39). Jerby-Arnon immune resistance are the resistance program combining a gene set related to T cell exclusion, post treatment, and functional resistance (40). Roh immune score is defined by the genes set involved in immune activation associated with tumor rejection (41). Ock anti-CTLA-4 signature is derived from the expression of 105 genes associated with the response to immunotherapy (42). EaSIeR model is based on multi-task machine learning to predict different hallmarks of immune responses (43). All these transcriptomics signatures were calculated following the methodology and code in the original studies. The format of RNA-sequence data we used was TPM.

### 2.7 Prediction of sensitive drugs and tumor evolutionary status based on computing framework

We wished TILPI computing frameworks perform in predicting the sensitive drugs of individualized chemotherapy. This research was based on the R package called pRRophetic (44). The version of pRRophetic was published in 2016 including 251 types of drugs. The second algorithm to identify sensitive drugs was oncopredict (45). We used semi-inhibitory concentration (IC50) as the boundary to pick out sensitive drugs for different risk subgroups. The drugs with lower IC50 were sensitive for this subgroup. The sensitive drugs for a risk subgroup must meet 2 filters: the P value of the Wilcoxon test< 0.05 and the P value of Spearman correlation analysis< 0.05. Furthermore, we used the connectivity map (CMap) to identify sensitive drugs inhibiting up-regulated TMB-derived oncogenic genes (https://clue.io/). The research was conducted in 28 cell lines, different doses (0.001 uM-90uM), and different processing times (1h-72h). We only selected known compounds and targets. And the absolute normalized CMap score of qualified drugs must be greater than 1.5. At last, we intersected 3 derived drugs to identify candidate drugs.

In addition, we analyze the modes of interaction between the candidate drugs and their targets based on Autodock Vina 1.2.2 (46). The molecular structures of candidate drugs were retrieved from PubChem (https://pubchem.ncbi.nlm.nih.gov/). And the 3D coordinates of their targets were downloaded from the PDB (http://www.rcsb.org/). Then we constructed the molecular docking models by Autodock Vina 1.2.2 (http://autodock.scripps.edu/).

Based on network pharmacology, we next searched for potential targets of candidate drugs targeting LUAD. LUAD targets are from the GeneCards database (www.genecards.org/). The SMILE numbers of the candidate drugs were acquired from the Pubchem database (https://pubchem.ncbi.nlm.nih.gov/) and then sequentially imported into the SwissTargetPrediction database (http://www.swisstargetprediction.ch/) for target prediction. Targets with a probability >0 were selected as potential targets for candidate drugs. The BATMAN-TCM database was also used to obtain the target information for candidate drugs (http://bionet.ncpsb.org.cn/batman-tcm/). The filter was score cutoff >10 and adjusted P_value >0.05. We also used NCI-60 cell line set in CellMiner database to search for genes associated with drug candidates (P<0.05) (47).

The stemness score is known as a significant score for the prediction of tumors’ progression (48). The epigenetically regulated-mRNA expression-based stemness score (EREG-mRNAss) was used to assess the tumor evolutionary status because EREG-mRNAss was related to known tumor biological functions, therapy sensitivity, clinical characteristics, and tumor pathology. Furthermore, we obtained another similar stemness score called RNA expression-based stemness score (RNAss). We planned to connect TILPI computing frameworks with stemness scores to estimate the evolutionary status of the tumor. Therefore, we conducted the correlation analysis between TILPI and various stemness scores.

### 2.8 Statistical analysis

We used R version 4.1.1 to analyze data and create figures and tables (https://www.r-project.org). We also drew diagrams with the help of an online website called bioinformatics (https://www.bioinformatics.com.cn/). The KM method was conducted to verify clinical characteristics’ independence of computing frameworks. The log-rank test was used to calculate the P value of survival difference between two subgroups. Furthermore, we used the Chi-Square test to prove clinical characteristics’ relationship to the computing framework. The other correlation analyses were based on the Wilcoxon test and Pearson correlation coefficient.

## 3 Results

### 3.1 Identify the TMB-derived immune lncRNA set

**Figure 2:**
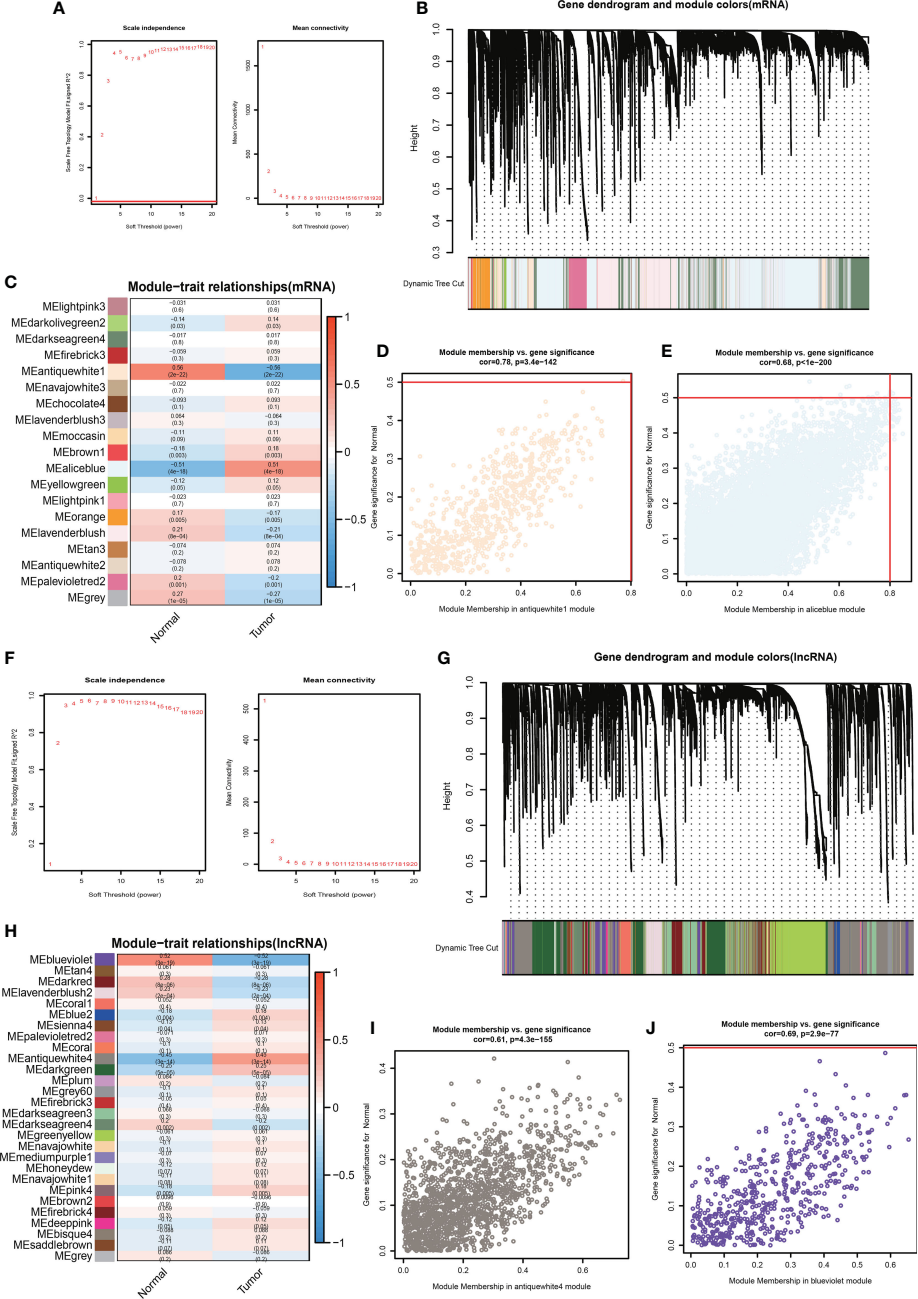
Determine the optimal power value (β = 4) taking into account both “scale independence” and “mean connectivity” **(A)**. There were 19 MEs according to lncRNAs’ functional relevance **(B, C)**. Verify the correlation between GS and MM. **(F)** The best power value (β) was 3 **(D, E)**. There were 28 MEs **(G, H)**. Verify the correlation between GS and MM **(I-J)**.

We collected 509 patients with TMB values and divided them into four equal parts according to the TMB score. Then we took the first 127 and the last 127 patients as the low TMB subgroup and high TMB subgroup. Furthermore, we conducted WGCNA to find TMB-derived mRNA modules, so the mRNA expressed differently between the high and low TMB subgroups was identified. We used Topological Overlap Matrix (TOM) to construct a new neighborhood matrix to reduce error and false correlation. Consequently, we sorted out the mRNA matrix and determined the optimal power value (β = 4). This value considered both scale independence and mean connectivity (**Figure 2A**). Moreover, we divided all mRNAs into 19 module eigengenes (MEs) based on the functional correlation (**Figures 2B, C**). In the end, we obtained 2 MEs expressing differently between the high and low TMB subgroups. They were MEantiquewhite1 (cor = -0.56, P = 2e-22) and MEaliceblue (cor = 0.51, P = 4e-18) (**Figure 2C**). However, the other 17 MEs were lower associated with the high TMB group (cor<0.4) (**Figure 2C**). Subsequently, we aimed to confirm the correlation between gene significance (GS) and module membership (MM). We got excellent results for MEantiquewhite1 (cor = 0.68, P< 1e-200) and MEaliceblue (cor = 0.78, P = 3e-142) (**Figures 2D, E**). But in the rest of the MEs, GS had poor correlations with MM. In conclusion, we obtained 8527 TMB-derived mRNAs from MEantiquewhite1 and MEaliceblue. Moreover, we used WGCNA to find TMB-derived lncRNA modules. And TOM was used to construct a new neighborhood matrix to reduce errors and false correlations. Lastly, we made the lncRNA matrix and determined the optimal power value (β = 3). This value considered both scale independence and mean connectivity (**Figure 2F**). Afterward, according to the functional relevance, we divided all lncRNAs into 28 MEs (**Figures 2G, H**). Finally, we found that two lncRNA MEs were correlated with TMB. They were MEblueviolet (cor=-0.52, P=3e-19) and MEantiquewhite4 (cor=0.45, P=3e-14) (**Figure 2H**). And the remaining 26 MEs were irrelevant (cor<0.4). Then, we intended to confirm the correlation between GS and MM. MEblueviolet (cor=0.69, p=2.9e−77) and MEantiquewhite4 (cor=0.61, p=4.3e−155) also did good jobs (**Figures 2I, J**). However, in the rest 26 MEs, GS was not highly correlated with MM. Finally, 2053 TMB-derived lncRNAs were identified based on MEblueviolet and MEantiquewhite4.

**Figure 3:**
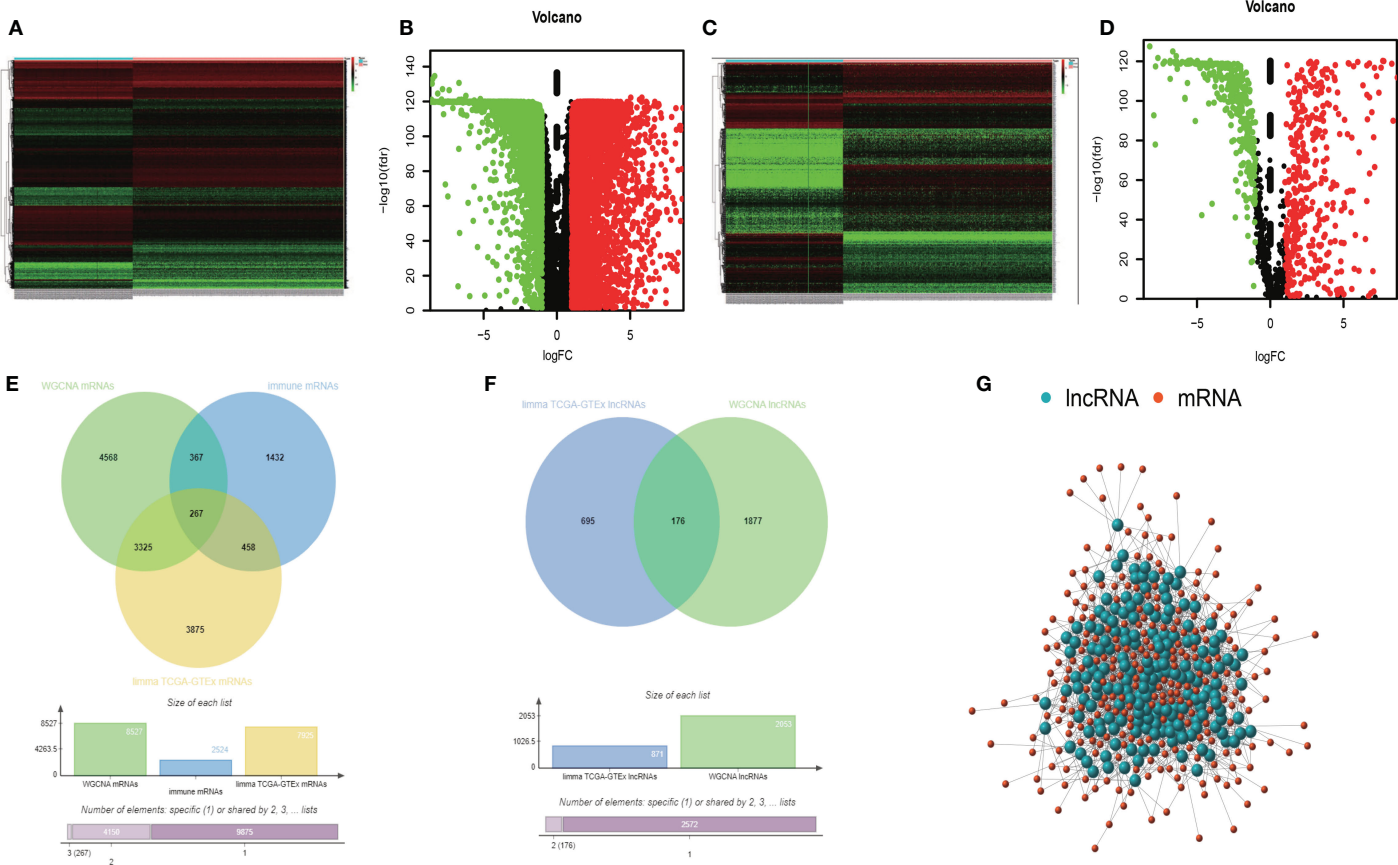
The heatmap of differently expressed mRNAs **(A)**. The volcano map of differently expressed mRNAs **(B)**. The heatmap of differently expressed lncRNAs **(C)**. The volcano map of differently expressed lncRNAs **(D)**. The intersection analysis of TMB-derived mRNAs, oncogenic mRNAs, and immune- related mRNAs **(E)**. The intersection analysis of TMB-derived lncRNAs and oncogenic lncRNAs **(F)**. The Pearson correlation network between lncRNAs and mRNAs **(G)**.

Furthermore, we used the limma package of R to identify differentially expressed mRNAs between 288 normal lung samples from GTEx and 513 LUAD samples from TCGA. Two prerequisites were required before confirming the differential expression of mRNAs: (1) |*log*_2_*FC*| ≥ 1 (2) FDR adjusted P value< 0.05. Then, we got a total of 14437 differentially expressed mRNAs. But only 7925 mRNAs met the above two prerequisites (**Figures 3A, B**). In addition, also for those samples, we found 1187 lncRNAs differentially expressed by limma package. Then we sorted out 871 oncogenic lncRNAs that met the abovementioned premises (**Figures 3C, D**).

Furthermore, we collected 2524 immune-related mRNAs from the immunology database and analysis portal (ImmPort) and systems biology of the innate immune response (InnateDB). Based on the above, we obtained 8527 TMB-derived mRNAs by WGCNA, 7925 oncogenic mRNAs selected by the limma package, and 2524 immune-related mRNAs. At last, we intersected these three mRNA sets and obtained 267 immune TMB-derived oncogenic mRNAs (**Figure 3E**). As for lncRNA, we identified 871 oncogenic lncRNAs and 2053 TMB-derived lncRNAs. Then we analyzed the intersection and got 176 eligible TMB-derived oncogenic lncRNAs (**Figure 3F**). Then we conducted the Pearson correlation analysis to investigate their correlation (**Figure 3G**). Finally, we obtained 36 immune TMB-derived oncogenic lncRNAs (cor>0.4), which was called TMB-derived immune lncRNA set (TILncSet). And the 43 immune TMB-derived oncogenic mRNAs (cor>0.4) were called TMB-derived immune mRNA set (TImSet). The TMB-derived immune gene set (TIgeneSet) consisted of TILncSet and TImSet.

### 3.2 Explore the functional biological pathways of TIgeneSet

**Figure 4:**
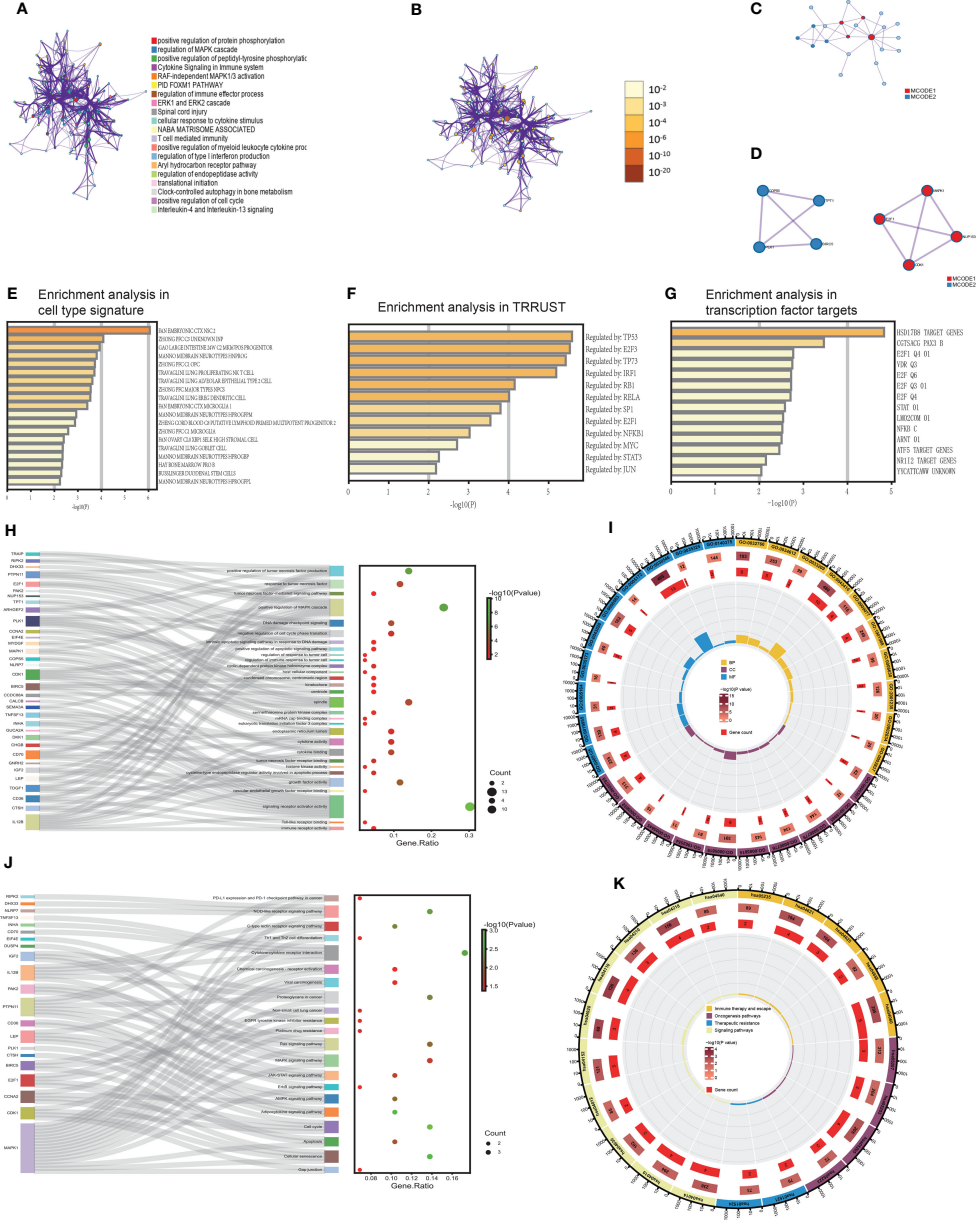
The functional biological pathways were clustered **(A, B)**. The PPI network and MCODE algorithm of TIgeneSet **(C, D)**. The enrichment analysis in cell type signature **(E)**. The enrichment analysis of TRRUST. **(G)** The enrichment analysis in transcription factor targets **(F)**. The GO functional enrichment analysis of TIgeneSet **(H, I)**. The KEGG functional enrichment analysis of TIgeneSet **(J, K)**.

As we all know, although lncRNA can’t encode proteins, it has immeasurable effects on cellular life activities. We intended to examine what the 43 mRNAs and 36 lncRNAs from these experiments would play in LUAD. Therefore, we utilized a meta scape to explore the potential functions of these 79 genes. The meaningful enriched pathways must meet the following prerequisites: P value<0.01, a minimum count of 3, and an enrichment factor >1.5. Furthermore, we made a network of enriched terms based on 20 pathway clusters with the smallest P value. In the network, each gene of pathway clusters was represented by a node and was colored by pathway cluster ID (**Figure 4A**). The size of nodes referred to gene counts of pathway clusters, and the color was correlated with the P value (**Figure 4B**). The function of 20 pathway clusters was brilliant, which included positive regulation of protein phosphorylation, regulation of MAPK cascade, cytokine signaling in the immune system, regulation of immune effector process, ERK1 and ERK2 cascade, T cell-mediated immunity, translational initiation, positive regulation of cell cycle.

Wielding the above genes, we also constructed a protein-protein interaction (PPI) network based on three pathways with the smallest P value (**Figure 4C**). Their functional descriptions were positive protein phosphorylation, regulation of MAPK cascade, and RAF-independent MAPK1/3 activation. If a subset contains between 3 and 500 proteins, we will conduct the Molecular Complex Detection (MCODE) algorithm to show its density in the network. The PPI network showed the relationship between 22 protein subsets of 3 pathways. MCODE1 represented the four red high-density protein subsets, and the other four blue high-density protein subsets were represented by MCODE2 (**Figure 4C**). MCODE1 included E2F1, MAPK1, CDK1, and NUP153. These four proteins possessed significant interactions between the two (**Figure 4D**). And MCODE2 included COPS5, TPT1, BIRC5, and PLK1. Except for the fact that there was no interaction between TPT1 and BIRC5, the rest of the proteins had functional interactions between them (**Figure 4D**). Backing to the macroscopic level, MCODE1 and MOCDE2 also interacted through 4 proteins (**Figure 4D**).

The enrichment analysis in cell type signature showed the relationship between 76 genes and some cell types, such as lung proliferating NK T cells and lung goblet cells. Lung proliferating NK T cells were associated with the innate form of the immune barrier. And the abnormal proliferation of lung goblet cells refers to LUAD (**Figure 4E**). The relationship between these genes and LUAD was inseparable. In addition, the enrichment analysis in transcriptional regulatory relationships unraveled by sentence-based text mining (TRRUST) indicated the relationship between 76 genes and transcription factors. These transcription factors or target genes corresponding to transcription factors were TP53, E2F3, TP73, IRF1, RB1, RELA, SP1, E2F1, NFKB1, MYC, STAT3, and JUN (**Figure 4F**). The above were sorted by P value. TP53 is closely related to the functional activity of LUAD cells. TP73 is highly homologous to TP53, its function involves all aspects of cellular life activities, and its transcriptionally translated protein p73 is a carcinostatic factor. IRF1 negatively regulated the expression of the oncogene kpna2 in LUAD cells under conditions of growth stimulation and hypoxia. SP1 is associated with LUAD transfer. E2F1 and KLF6 form a positive feedback pathway in LUAD, regulating the cell cycle and leading to cisplatin resistance in LUAD. MYC drives the evolution of small-cell lung cancer subtypes. Activating the STAT3 signaling pathway can promote the development of LUAD. Therefore, the relationship between these transcription factors or target genes and LUAD is indivisible.

Furthermore, the enrichment analysis in transcription factor targets showed 14 connected targets (**Figure 4G**). The E2F family is involved in developing LUAD and affects prognosis and efficacy. E2F1 is correlated with the cell cycle and LUAD resistance. Abnormalities in the STAT pathway are closely related to cell hyperplasia, differentiation, and LUAD development.

We manipulated the GO and KEGG functional enrichment analysis to explore further what roles 36 lncRNAs and 43 mRNAs play in LUAD. P value<0.05 was the filter in GO and KEGG analysis. Then we found 961 meaningful pathways in the GO analysis. There were 827 pathways for biological process (BP), 51 for cell component (CC), and 83 for molecular function (MF). We respectively selected ten excellent pathways in BP, CC, and MF to draw intuitive diagrams. Pathways of BP were associated with the regulation of tumor cell response, apoptosis pathway, cell cycle, tumor-related signaling pathway, and regulation of tumor necrosis factor. Pathways of CC were associated with mitotic processes, protein translation processes, and protein kinase complexes. Pathways of MF were associated with immune-related receptors, cytokines, protein kinases, their related functions, and regulation of growth factors. Then we obtained a diagram that showed the correlation between functional pathways and genes. In the figure, each functional pathway was wired to the corresponding genes. In the adjacent dot plot, larger dots indicated more genes associated with the corresponding pathway, and the dots were colored according to the P value. The gene ratio was the ratio of the number of genes associated with the pathway to the total number of genes obtained (**Figure 4H**). Obviously, in BP, the positive regulation of the MAPK cascade was associated with the most genes. This pathway was associated with ten genes, which were TDGF1, LEP, IGF2, DHX33, CD36, DKK1, SEMA3A, PTPN11, MYDGF, and RIPK2. And other pathways, like a response to tumor necrosis factor and positive regulation of tumor necrosis factor production, were associated with 5 and 6 genes (**Figure 4H**). In CC, the spindle was connected with the most genes. They are PLK1, BIRC5, MAPK1, CDK1, ARHGEF2, and TPT1 (**Figure 4H**). In MF, signaling receptor activator activity was correlated with the most genes. These 13 genes were TDGF1, LEP, IGF2, GNRH2, IL12B, CD70, CHGB, DKK1, GUCA2A, INHA, TNFSF13, SEMA3A, and CALCB. In conclusion, a sector chart was constructed to show the results visually (**Figure 4I**).

We got 46 functional pathways in the KEGG analysis and selected 21 significant ones. These functional pathways were divided into immune therapy and escape, oncogenesis, therapeutic resistance, and signaling pathways. And there were relationships between functional pathways and genes (**Figure 4J**). Each pathway was wired to the associated genes (**Figure 4J**). In immune therapy and escape, cytokine-cytokine receptor interaction was associated with five genes, including LEP, IL12B, CD70, INHA, and TNFSF13. And NOD-like receptor signaling pathway possesses four corresponding genes. There are NLRP7, DHX33, MAPK1, and RIPK2 (**Figure 4J**). The number of genes associated with this pathway was similar to oncogenesis pathways. The four genes related to proteoglycans in cancer were IGF2, MAPK1, IL12B, and PTPN11. We also found four genes associated with the cell cycle, which included PLK1, CCNA2, CDK1, and E2F1. CCNA2, MAPK1, CDK1, and E2F1 were enriched in cellular senescence (**Figure 4J**). In therapeutic resistance, we proved that these genes took part in both EGFR tyrosine kinase inhibitor resistance and platinum drug resistance (**Figure 4J**). In signaling pathways, the RAS signaling pathway and MAPK signaling pathway were also enriched pathways (**Figure 4J**). Moreover, we drew a sector chart to show the results visually (**Figure 4K**).

### 3.3 Construction and validation of the TILPI computing framework

3.3.1 Construct the TILPI computing framework in the training group

**Figure 5:**
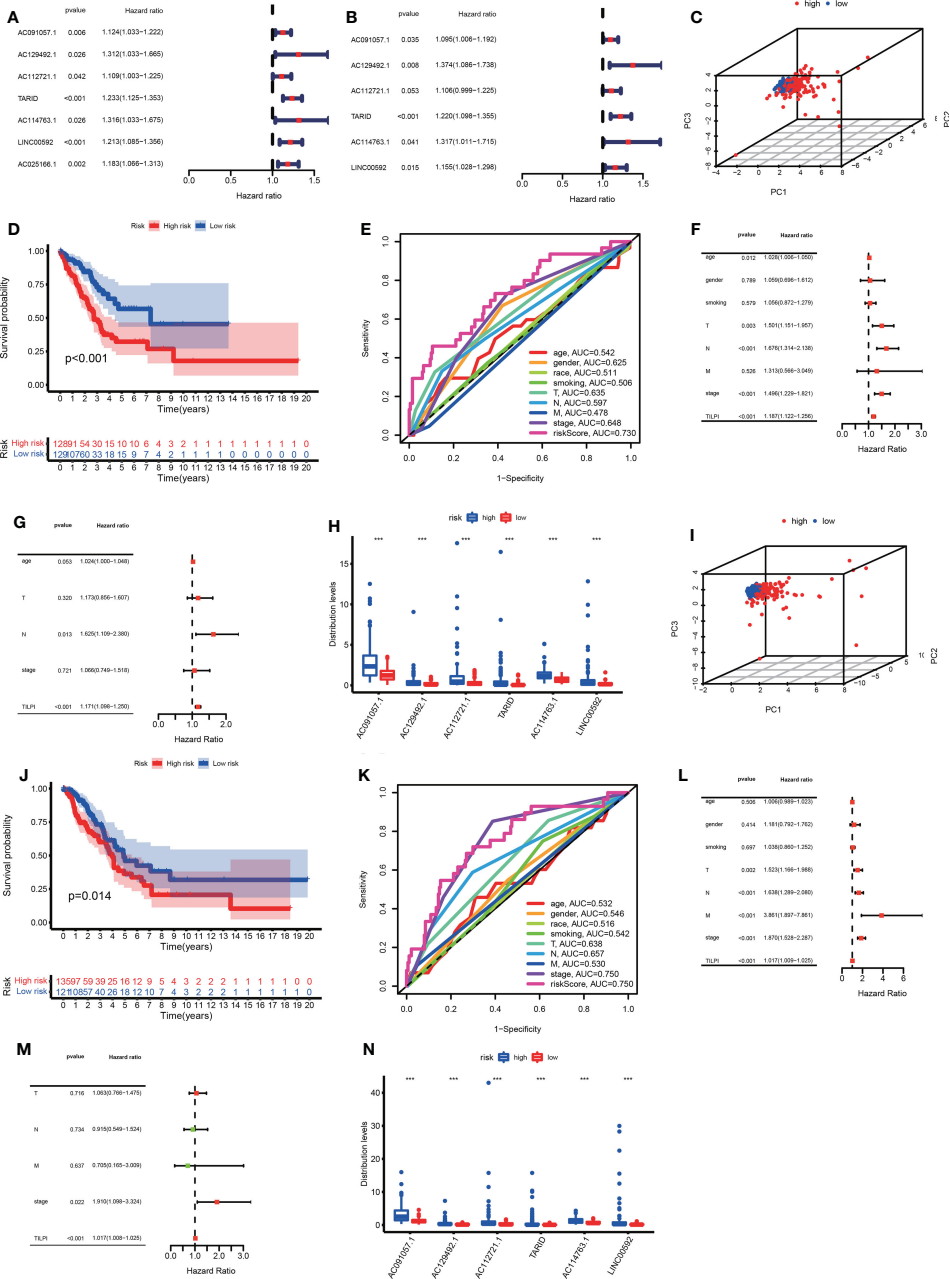
We successfully identified TILncSig based on univariate and multivariate Cox proportional hazards regression analyses in the training group **(A, B)**. In the training group, the 3D PCA, survival analysis, and ROC curve verify the reliability of the TILPI computational framework **(C-E)**. The univariate and multivariate Cox analyses prove the independent prognostic hazard of TILPI **(F, G)**. The expression of TILncSig was different in different risk subgroups **(H)**. Verify the reliability of the TILPI computational framework in the testing group **(I-N)**. ***P < 0.001.


To enhance the practicability of TILncSet, we hoped it could determine the prognosis of patients with LUAD. According to the above results, we knew that TILncSet possibly induces LUAD, which is more likely to participate in the pathogenic pathway of LUAD than other lncRNAs. Therefore, we sought prognostic lncRNA signatures of LUAD based on TILncSet. We divided all meaningful TCGA samples (n=513) (the TCGA group) into two groups, namely the training group (n=257) and the testing group (n=256) (**Table S1**). We counted some clinical characteristics of patients in each group, and it was easy to see that these clinical characteristics were particularly evenly distributed in each group, so the reliability of the grouping was preliminarily verified (**Table S1**). To screen for prognostic-related lncRNAs, univariate Cox proportional hazard regression analysis was used to analyze the relationship between expression levels of 36 TMB-derived immune lncRNAs and OS in the training group, and 7 TMB-derived immune lncRNAs were found to be significantly associated with the prognosis of LUAD patients (AC091057.1, AC129492.1, AC112721.1, TARID, AC114763.1, LINC00592, AC025166.1) (**Figure 5A**). In addition, we hoped to screen out lncRNAs with independent prognostic value from these 7 candidate lncRNAs, and conducted multivariate Cox proportional hazards regression analysis for these 7 candidate lncRNAs. Finally, 6 of 7 candidate lncRNAs (AC091057.1, AC129492.1, AC112721.1, TARID, AC114763.1, LINC00592) were identified as independent prognostic lncRNAs (**Figure 5B**). The six all lncRNAs of TILncSig were risk factors for LUAD patients because their coefficients based on multi-Cox analysis were all positive. Then a TMB-derived immune lncRNA signature (TILncSig) was constructed. Next, we built a computing framework to evaluate the risk score generated by TILncSig’s expressions in individuals. The computing framework was as follows:

$$\text{TILPI}=\text{exp}\left(\text{ln}\left(\text{h}\left({\text{t}}_{0}\right)\right)+\sum_{\text{i}=1}^{\text{n}}\text{coef}\left({\text{lncRNA}}_{\text{i}}\right)*\text{expr}\left({\text{ lncRNA}}_{\text{i}}\;\right)\right)$$

ln(*h*(*t*_0_))=-0.7850

TILPI is a prognostic risk score for the LUAD patients. For each individual, his TILPI = exp (-0.7850 + 0.0908 * expression (AC091057.1) + 0.3175 * expression (AC129492.1) + 0.1009 * expression (AC112721.1) + 0.1987 * expression (TARID) + 0.2754 * expression (AC114763.1) + 0.1442 * expression (LINC00592)). The median score of the LUAD patients in the training group (median =0.8510) was used as a risk cutoff to classify patients into the low-risk group with low TILPI (TILPI ≤0.8510) or high-risk group with high TILPI (TILPI >0.8510).

To further verify the effectiveness of grouping, we utilized 3D principal component analysis (3D PCA) to verify the reliability in the training group, and the results proved that our grouping was reliable (**Figure 5C**). Kaplan–Meier analysis showed that the survival time of patients in the low-risk group are significantly better than patients in the high-risk group (P<0.001) (**Figure 5D**). In the training group, the 5-year survival rate of the high-risk subgroup was 7.8%, worse than 11.6% in the low-risk subgroup. Based on the above data, we demonstrated that TILPI did a good job of relating to the OS of patients. To prove the credibility of TLIPI, we used the time-dependent ROC curves to observe the training group. Finally, the area under the curve (AUC) value of the TILPI was 0.730 in the training group. It meant that TILPI had excellent credibility in judging OS in the training group, but other clinical characteristics didn’t (age (ACU=0.542), gender (ACU=0.625), race (AUC=0.511), smoking (AUC=0.506), pathological stage (AUC=0.648), pathological T (AUC=0.635), pathological N (AUC=0.597), pathological M (AUC=0.478)) (**Figure 5E**).

We also used univariate Cox proportional hazards regression analysis to verify the independence of clinical characteristics (age, gender, smoking, pathological stage, and pathological TNM) and TILPI in the training groups. The results showed that age, pathological stage, pathological T, pathological N, and TILPI were independent risk factors (P<0.05), while gender, smoking, and pathological M wasn’t (P>0.05) (**Figure 5F**) (**Table S2**). Next, we used multivariate Cox proportional hazards regression analysis on age, pathological stage, pathological T, pathological N, and TILPI. In the end, only pathological N and TILPI were eligible independent risk factors in the training group (P<0.05) (**Figure 5G**) (**Table S2**). We considered that the expressions of TILncSig were factors that affected individual TILPI. Firstly, all of TILncSig were expressed less in the low-risk subgroup than in the high-risk subgroup (P<0.001) (**Figure 5H**). Therefore, AC091057.1, AC129492.1, AC112721.1, TARID, AC114763.1, and LINC00592 were likely disadvantageous to LUAD patients in the training group.

3.3.2 Verify the reliability of the TILPI computing framework in the TCGA group and the testing group

In the previous experiment in the training group, the potential of the computing framework to relate to OS was demonstrated. However, it was still necessary to further verify its reliability in the testing group. When the same TILncSig and risk cutoff as those derived from the training group was applied to the testing group, 256 patients of the testing group was classified into the low-risk group (n = 121) and high-risk group (n = 135) with significantly different overall survival. As **Figure 5J** showed that the overall survival of 135 patients in the high-risk group was much poorer than 121 patients in the low-risk group (P=0.014). Of course, we also used 3D PCA to verify the reliability of grouping in the testing group. Meanwhile, the result proved that our grouping was reliable (**Figure 5I**). The time-dependent ROC curves showed that TILPI (AUC=0.750), pathological stage (AUC=0.750), and pathological N (AUC=0.657) had credibility in judging OS in the testing group, but other clinical characteristics didn’t [age (ACU=0.532), gender (ACU=0.546), race (AUC=0.516), smoking (AUC=0.542), pathological T (AUC=0.638), pathological M (AUC=0.530)] (**Figure 5K**). Furthermore, we verified the independence of clinical characteristics and TILPI in the testing groups. And the result showed that only the pathological stage and TILPI were statistically meaningful (P<0.05) (**Figures 5L, M**) (**Table S2**). Moreover, the expressions of TILncSig influencing TILPI in the testing group were similar to the training group. AC091057.1, AC129492.1, AC112721.1, TARID, AC114763.1, and LINC00592 were all life-threatening in the testing group, too (P<0.001) (**Figure 5N**).

**Figure 6:**
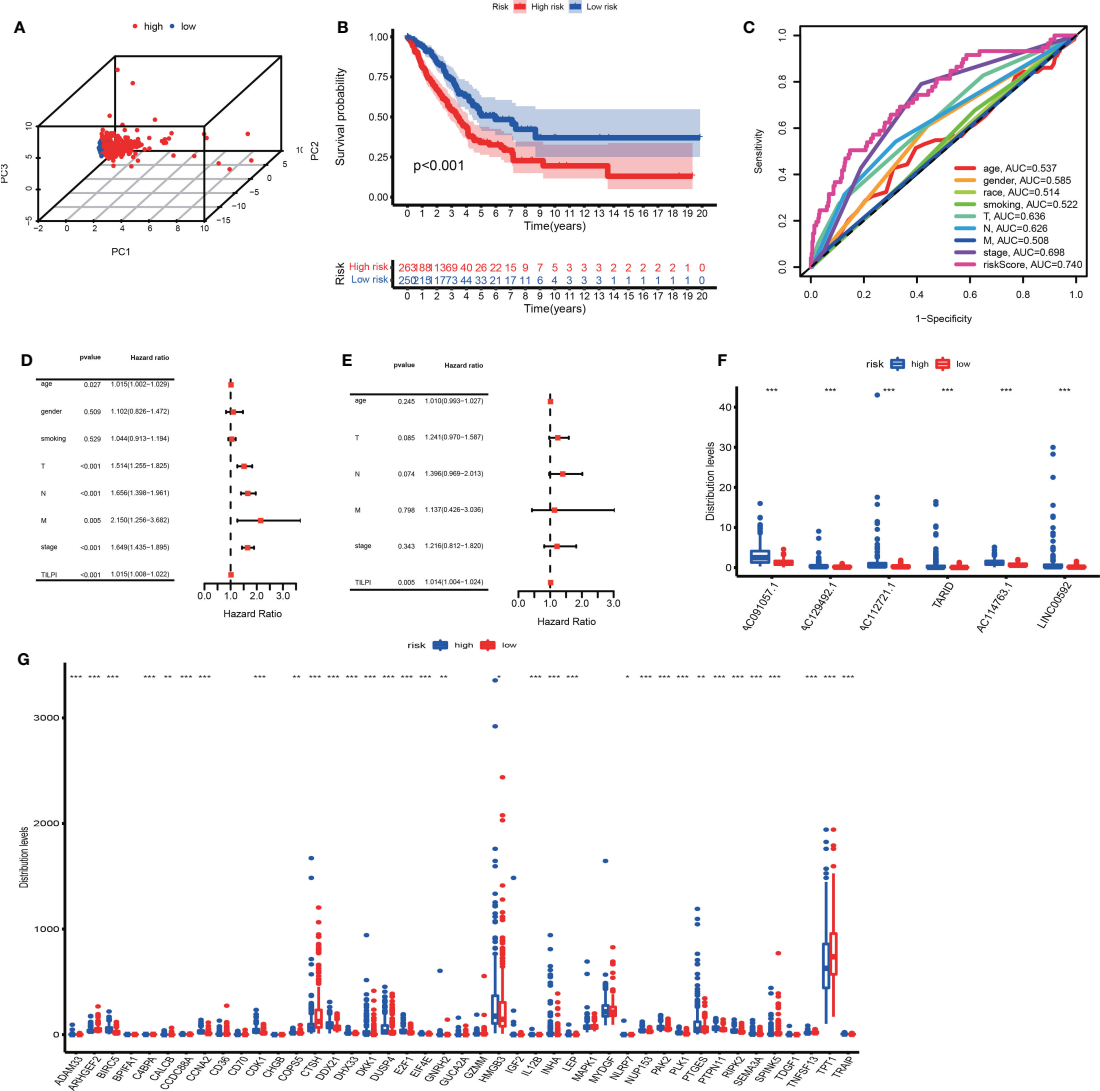
Verify once again the reliability of the TILPI computational framework in the TCGA group **(A-G)**. *P < 0.05; **P < 0.01; ***P < 0.001.

The prognostic performance of the TILncSig in the TCGA group was similar to the above results. We similarly used the median TILPI of the training group (0.8510) as a cutoff to divide the TCGA group (n=513) into the low-risk subgroup (n=250) and high-risk subgroup (n=263). As we expected, 3D PCA also showed that grouping based on TILPI was reliable (**Figure 6A**). The overall survival of 263 patients in the high-risk group was much poorer than 250 patients in the low-risk group (P<0.001) (**Figure 6B**). And Similar to the training group, the survival rate was 9.9% in the high-risk group at 5 years lower than 13.2% in the low-risk group (**Figure 6B**). The results of the time-dependent ROC curves in the TCGA group showed that TILPI (AUC=0.740) and pathological stage (AUC=0.698) had credibility in judging OS in the TCGA group while other clinical characteristics didn’t (age (ACU=0.537), gender (ACU=0.585), race (AUC=0.514), smoking (AUC=0.522), pathological T (AUC=0.636), pathological N (AUC=0.626), pathological M (AUC=0.508)) (**Figure 6C**). Furthermore, according to the independence analyses based on univariate and multivariate Cox proportional hazards regression of clinical characteristics and TILPI in the TCGA group, the only meaningful result was TILPI (P=0.005) (**Figures 6D, E**) (**Table S2**). In addition, we also demonstrated that AC091057.1, AC129492.1, AC112721.1, TARID, AC114763.1, and LINC00592 were all life-threatening in the TCGA group too (P<0.001) (**Figure 6F**).

Unlike the other two groups, we investigated whether the expressions of 43 above mRNAs affected the individual TILPI in the TCGA group. We found that 30 of the 43 mRNAs were associated with TILPI. The 21 mRNAs were expressed more in the high-risk subgroup (BIRC5, CCDC88A. CCNA2, CDK1, CALCB, COPS5, DDX21, DHX33, DKK1, DUSP4, E2F1, EIF4E, HMGB3, INHA, LEP, NLRP7, NUP153, PAK2, PLK1, PTGES, PTPN11, RIPK2, SEMA3A, TRAIP). And nine mRNAs were expressed more in the low-risk subgroup (ADAM33, ARHGEF2, C4BPA, CTSH, GNRH2, IL12B, SPINK5, TNFSF13, TPT1) (P<0.05) (**Figure 6G**). These results suggested that the expressions of 9 mRNAs would improve the prognosis of LUAD patients, while 21 other mRNAs were adverse.

Subsequent survival analyses examined the likelihood of a single gene predicting survival. AC114763.1, LINC00592, TARID, and AC091057.1 independently predicted patient survival (P<0.05) (**Figures S1A-D**). AC129492.1 and AC112721.1, on the other hand, did not perform well (**Figures S1E, F**). TILPI’s potential to predict survival was also extended to 1077 patients with NSCLC. Taking the median TILPI (0.8510) of the above training group as cutoff, survival probability of patients in the high TILPI group was significantly lower than those in the low TILPI group (P=0.01) (**Figure S1G**).

At last, we tried to verify the reliability of TILPI in GEO datasets and collected 9 LUAD datasets with OS (1644 samples). They were GSE11969, GSE13213, GSE26939, GSE31210, GSE36471, GSE63459, GSE68465, GSE68571, and GSE72094. However, the platforms of 9 all external datasets didn’t cover TILncSig.

#### 3.3.3 Clinical characteristics independence analysis of TILPI

**Figure 7:**
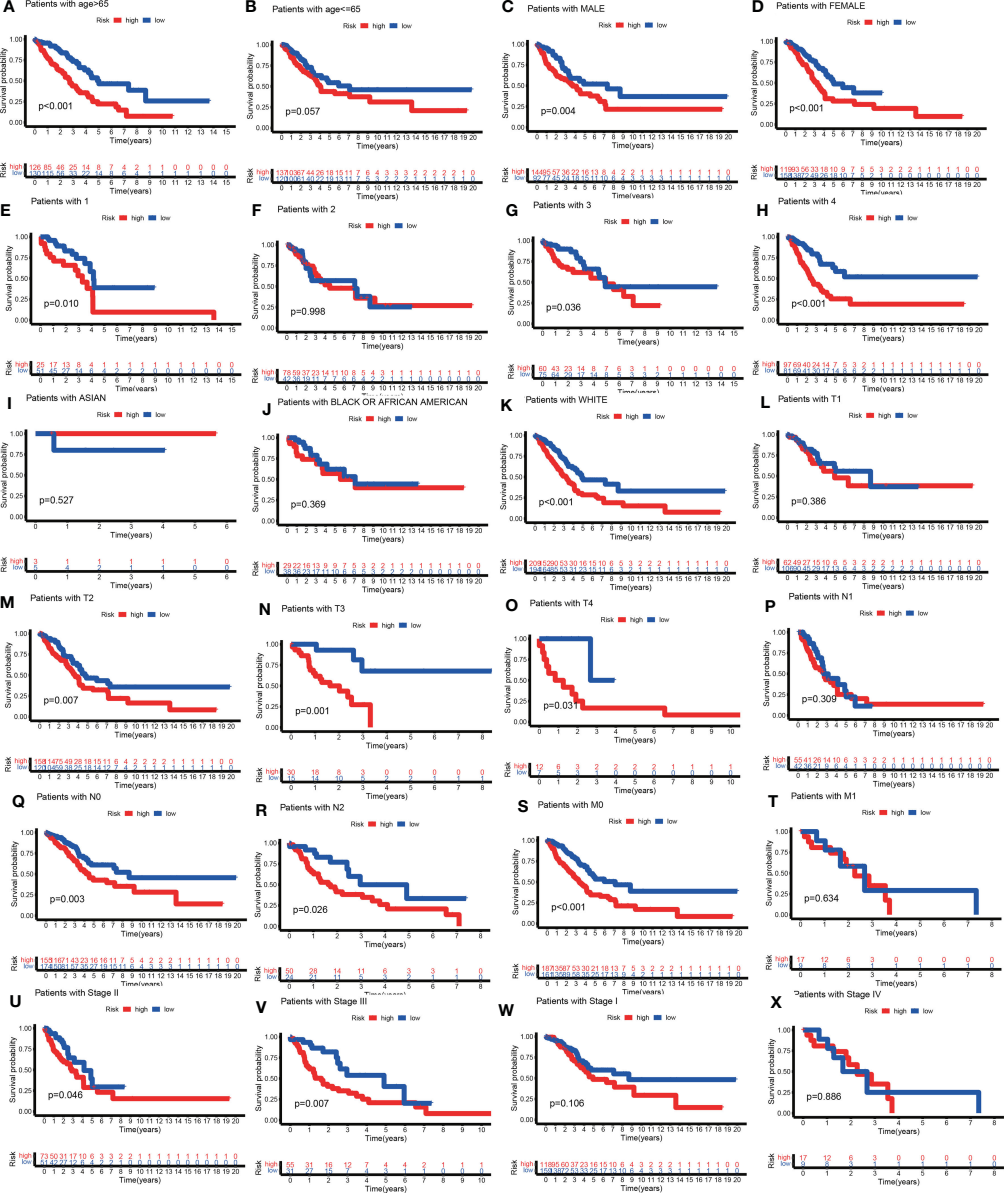
Explore the clinical independence of the TILPI computational framework by grouping. The low-risk and the high-risk subgroups exhibited obvious survival differences in the old subgroup **(A)**, while these differences weren’t statistical meaning in the young subgroup **(B)**. For the gender, there were survival differences between the high-risk and low-risk subgroups in the male samples **(C)**, while the female samples were as well **(D)**. But TILPI wasn’t independent of smoking **(E-H)** and race **(I-K)**. TILPI was unable to relate to the survival probability of patients in the T1 subgroup, but it was able to relate to the survival probability of patients in the T2, T3, and T4 subgroups **(L-O)**. In addition, TILPI was not independent in the N1 subgroup **(P)**, but it was independent in the N0 and N2 subgroups **(Q, R)**. What is more, TILPI was able to divide patients into high-risk and low-risk subgroups in the M0 subgroup **(S)**, while was not in the M1 subgroup **(T)**. Moreover, TILPI was valid in the stage II and stage III subgroups **(U, V)**, but it was invalid in the stage I and stage IV subgroup **(W, X)**.

Curious about whether the prognostic value of the TILPI was independent of common clinical characteristics, multivariate Cox regression analyses were performed on age, gender, the degree of smoking, race, pathological TNM and pathologic stage. (**Table S3**). Firstly, we divided samples based on age into the old subgroup (age > 65, n = 262) and the young subgroup (age ≤ 65, n = 260). As the figure showed that the low-risk subgroup and the high-risk subgroup exhibited obvious survival differences in the old subgroup (P< 0.001) (**Figure 7A**), while these differences weren’t statistical meaning in the young subgroup (P=0.057) (**Figure 7B**). For the gender, there were survival differences between the high-risk subgroup and low-risk subgroup in the male samples (n=242, P=0.004) (**Figure 7C**), while the female samples (n=280) were as well (P<0.001) (**Figure 7D**). But we found that TILPI wasn’t independent of smoking (P>0.05) (**Figures 7E-H**) or race (**Figures 7I-K**). Regarding the pathological T, the computing framework was unable to relate to the survival probability of patients in the T1 subgroup (n=172, P=0.386), but it was able to relate to the survival probability of patients in the T2 subgroup (n=281, P=0.007), T3 subgroup (n=47, P=0.001), and T4 subgroup (n=19, P=0.031) (**Figures 7L-O**). In addition, the computing framework wasn’t independent in the N1 subgroup (n=99, P=0.309) (**Figure 7P**), but it was independent in the N0 subgroup (n=335, P=0.003) and N2 subgroup (n=75, P=0.026) (**Figures 7Q, R**). What’s more, the computing framework was able to divide patients into high-risk subgroups and low-risk subgroups in the M0 subgroup (n=335) (P<0.001) (**Figure 7S**), while wasn’t in the M1 subgroup (n=26, P=0.634) (**Figure 7T**). Moreover, the computing framework was valid in the stage II subgroup (P=0.046) and stage III subgroup (P=0.007) (**Figures 7U, V**), but it was invalid in the stage I subgroup (P=0.106) or stage IV subgroup (P=0.886) (**Figures 7W, X**). In conclusion, there wasn’t independence between TILPI and seven clinical characteristics (age, smoking, race, pathological stage, pathological T, pathological N, and pathological M).

#### 3.3.4 Clinical characteristics correlation analysis of TILPI

**Figure 8:**
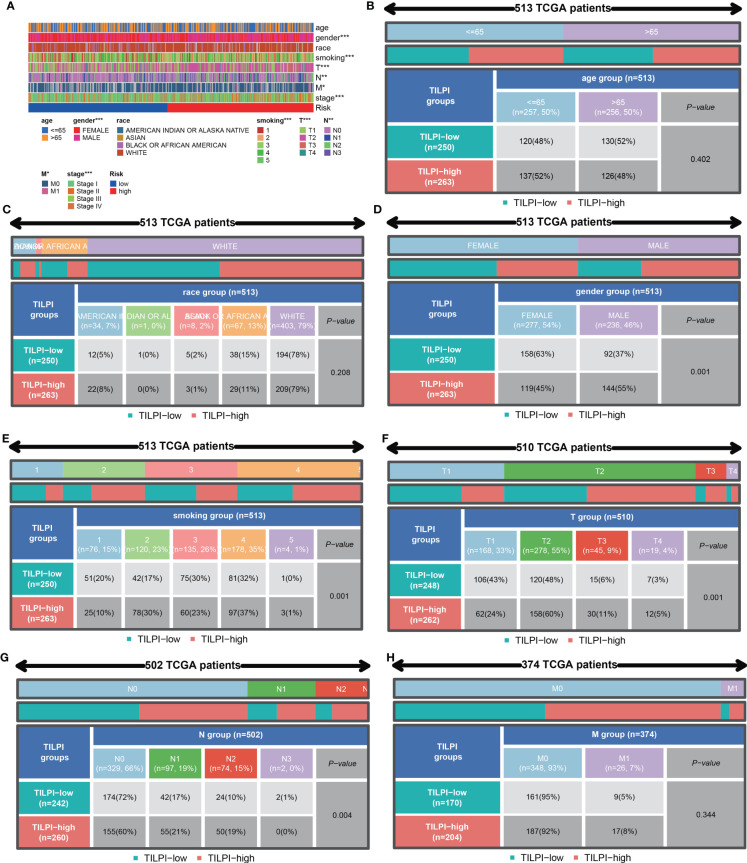
The correlation analyses between TILPI computational framework and clinical characteristics. TILPI was correlated with gender, smoking, pathological TNM, and pathological stage **(A)**. TILPI wasn’t associated with **(B)**, as was race **(C)**. The higher TILPI was more likely to occur in male patients **(D)**. TILPI was related to smoking **(E)**. What’s more, the correlation between TILPI and pathological T was statistically meaningful **(F)**, as was pathological **(G)**. But pathological M was not **(H)**. TILPI, TMB-derived immune lncRNA prognostic index. *P < 0.05; **P < 0.01; ***P < 0.001.

We were also curious about whether the prognostic value of the TILPI was associated with common clinical characteristics, Chi-square tests were performed on age, gender, the degree of smoking, race, pathological TNM and pathologic stage (**Figure 8A**) (**Table S3**). Firstly, it was obvious that the age of patients wasn’t associated with TILPI (P = 0.402) (**Figure 8B**) or race (P=0.208) (**Figure 8C**). There were more female patients than male patients, but higher TILPI was more likely to occur in male patients than in female patients (P = 0.001) (**Figure 8D**). Furthermore, we also found that TILPI was related to smoking (P = 0.001) (**Figure 8E**). What’s more, pathological T and pathological N were statistically meaningful (P = 0.001, P = 0.003) (**Figures 8F, G**), but pathological M was not (P=0.344) (**Figure 8H**). The pathological stage was closely related to TILPI. In conclusion, five clinical characteristics were associated with TILPI, which included gender, smoking, pathological stage, pathological T, and pathological N.

According to independence and correlation analysis, we knew that TILPI was related to various clinical characteristics (smoking, pathological stage, pathological T, and pathological N) (**Table S3**). Thus, the reliability of TILPI in predicting prognosis may depend on these clinical features.

#### 3.3.5 GSEA pathway correlation analysis of TILPI

**Figure 9:**
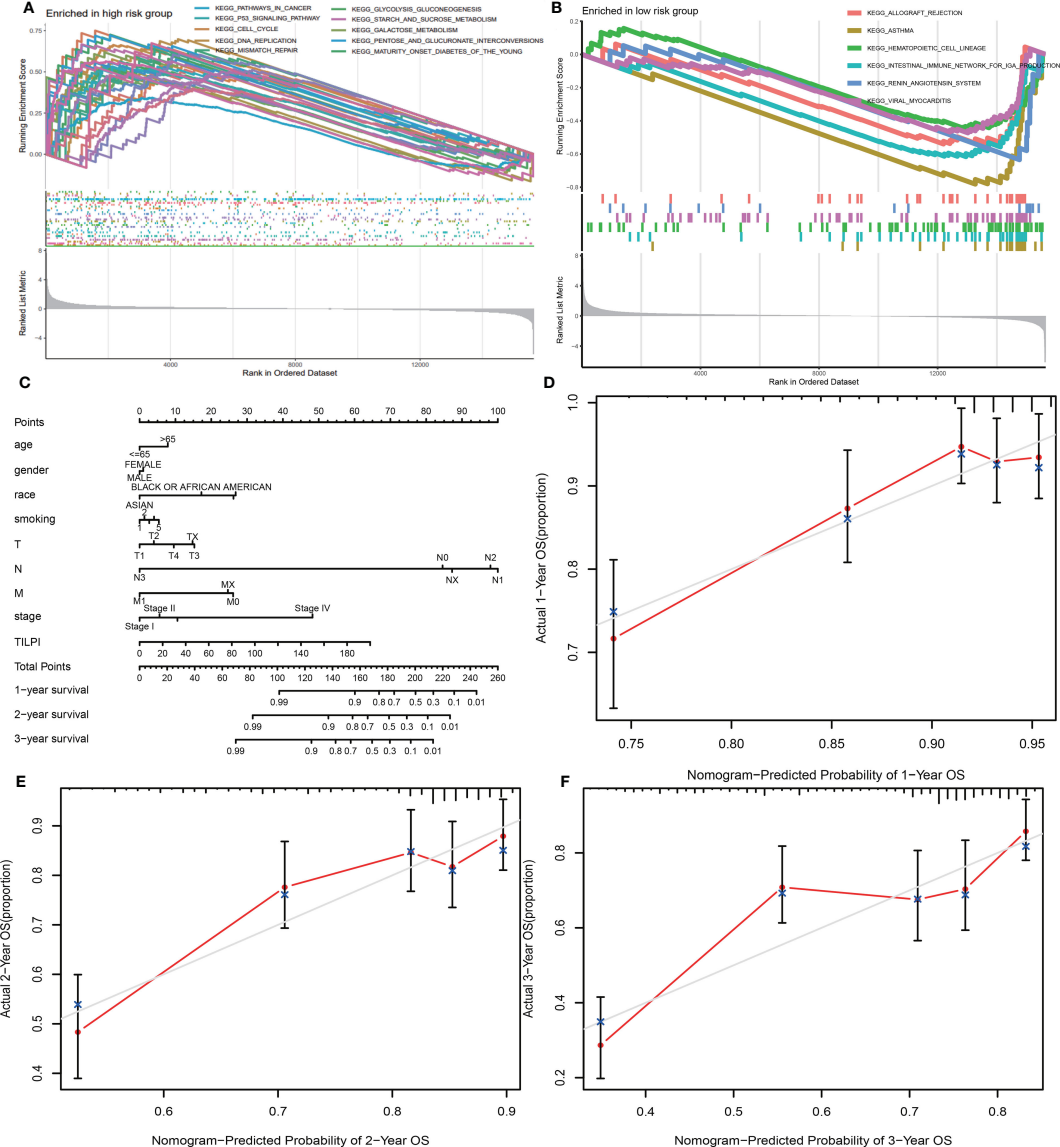
30 pathways were enriched in the high-risk group, and ten possessed excellent biological functions concerning LUAD **(A)**. There were six significant pathways enriched in the low-risk subgroup **(B)**. A new model was constructed consisting of TILPI and several clinical characteristics (age, gender, race, smoking, pathological stage, and pathological TNM) **(C)**. The new model’s 1, 2, and 3-year survival rate predictions fit well with the actual survival time **(D-F)**.

Through GSEA, we found 36 significant enriched pathways in different risk subgroups (P<0.05). Thirty pathways were enriched in the high-risk group. And ten pathways possessed excellent biological functions concerning LUAD (pathways in cancer, cell cycle, p53 signaling pathway, mismatch repair, DNA replication, starch and sucrose metabolism, glycolysis gluconeogenesis, galactose metabolism, pentose and glucuronate interconversions, maturity-onset diabetes of the young) (**Figure 9A**). In the high-risk group, five pathways were related to cell division and DNA mutations, while the last five pathways were related to energy metabolism, meaning we could kill LUAD cells by affecting their division and energy metabolism. Furthermore, six significant pathways enriched in the low-risk group (allograft rejection, asthma, hematopoietic cell lineage, intestinal immune network for IgA production, renin-angi system, viral myocarditis) (**Figure 9B**). In the low-risk group, three pathways were all immune response-activated pathways, suggesting that immune activation may be responsible for protecting the low-risk group. Therefore, individuals in the high-risk group may transform into the low-risk group by activating these immune pathways, thus prolonging OS.

#### 3.3.6 Construction of the prognosis nomogram based on TILPI and clinical features

To use TILPI more accurately to stratify the risk of LUAD patients in the clinic, we used TILPI and several clinical characteristics (age, gender, race, smoking, pathological stage, pathological TNM) to construct a new model that could calculate the OS probability (**Figure 9C**). To verify the reliability of this model, we fitted the calculated survival time with the actual survival time. The results showed that the 1-year survival rate prediction, 2-year survival rate prediction, and 3-year survival rate prediction all had a good fit with the actual survival time (**Figures 9D-F**). It also verified the reliability of TILPI. We confirmed that this new prognostic calculation model enabled to help clinicians to calculate the survival time of patients more easily.

### 3.4 Mapping of immune landscape based on TILPI

The tumor immune microenvironment (TIME) is the soil of immunotherapy. The ratio of immune cells will show more accurate treatment in individualized immunotherapy if the relationship between immune cells and TIME is close-knit. Therefore, we applied eight algorithms to describe the immune infiltration landscape in detail. These algorithms were cell type identification by CIBERSORT, CIBERSORT-ABS, EPIC, MCPCOUNTER, QUANTISEQ, TIMER, TISIDB, and XCELL. Firstly, we found 22 types of immune cells by CIBERSORT. And we drew four pictures that described the composition of immune cells. These plots were based on the Wilcoxon test and illuminated the difference between 22 immune cells in high-risk and low-risk subgroups.

**Figure 10:**
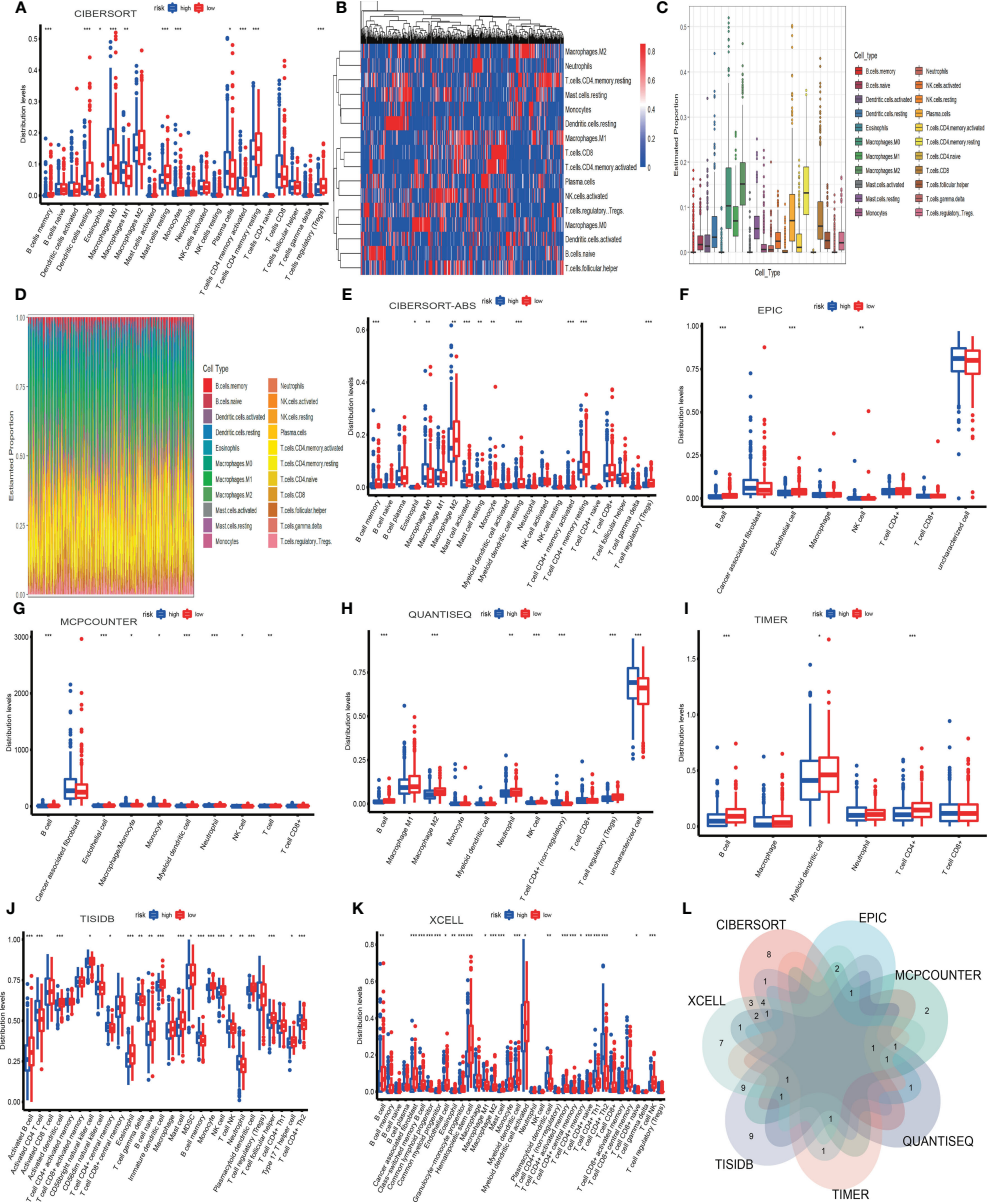
The infiltration landscape of 22 immune cells based on CIBERSORT **(A-D)**. The other seven algorithms showed the infiltration of immune cells, which included CIBERSORT-ABS, EPIC, MCPCOUNTER, QUANTISEQ, TIMER, TISIDB, and XCELL **(E-K)**. The intersection of immune cells of different algorithms **(L)**. *P < 0.05; **P < 0.01; ***P < 0.001.

Furthermore, we found that the distribution of 11 immune cells in the subgroups was statistically significant (P<0.05) (**Figure 10A**). There were four types of immune cells more distributed in the high-risk subgroup. They were macrophage M0, macrophage M1, plasma cell, and T cell CD4 memory activated. And the other seven types of immune cells were more distributed in the low-risk subgroup, which included B cell memory, dendritic cells resting, monocyte, mast cell resting, eosinophil, T cell CD4 memory resting, T cell regulatory (Tregs). However, the remaining 11 types of immune cells were not significantly statistical, which included B cell naive, dendritic cell activated, macrophage M2, mast cell activated, neutrophil, NK cell activated, NK cell resting, T cell CD4 naïve, T cell CD8, T cell follicular helper, T cell gamma delta. And the heatmap showed 16 types of immune cell expression. And the more immune cells are expressed, the more the color in the graph tends to be red.

Conversely, the fewer immune cells are expressed, the more the color in the heatmap inclines to be blue (**Figure 10B**). The plot showed Twenty-two types of immune cells and their expression. Macrophage M2 was the most expressed (**Figure 10C**). In the landscape diagram, the immune infiltration landscape of each sample was shown. Twenty-two colors represented twenty-two types of cells, the abscissa represented the samples, and the percentage of color length on the ordinate represented the ratio of immune cells (**Figure 10D**). Secondly, we wielded CIBERSORT-ABS to obtain a picture based on the Wilcoxon test, which indicated the distribution of 22 types of immune cells in the high-risk and low-risk subgroups (**Figure 10E**).

Furthermore, we found that the graph’s distribution of 11 immune cells was statistically significant (P<0.05). They were three types more distributed in the high-risk subgroup. They were macrophage M0, mast cell resting, and T cell CD4 memory activated. The other eight types were more distributed in the low-risk subgroup, which involved B cell memory, eosinophil, macrophage M2, mast cell activated, monocyte, myeloid dendritic cell resting, T cell CD4 memory resting, T cell regulatory (Tregs). But the remaining ten types were not statistically significant, which included B cell naive, B cell plasma, macrophage M1, myeloid dendritic cell activated, neutrophil, NK cell activated, NK cell resting, T cell CD4 naive, T cell CD8, T cell follicular helper.

Thirdly, we applied EPIC to obtain a graph based on the Wilcoxon test, which implied the distribution of 8 types of immune cells in high-risk and low-risk subgroups. (**Figure 10F**) Furthermore, we found that graph’s distribution of three immune cells was statistically significant (P<0.05). And there was 1 type more distributed in the high-risk subgroup. It was the NK cell. The other two types were more distributed in the low-risk subgroup, B cell and endothelial cell. But the remaining five types were not statistically significant, including CAF, macrophage, T cell CD4, T cell CD8, and uncharacterized cells. Fourthly, we used MCPCOUNTER to draw a picture based on the Wilcoxon test, which showed the distribution of 10 types of immune cells in high-risk and low-risk subgroups (**Figure 10G**).

Furthermore, we found that CAF and T cell CD8 were not statistically significant, and the distribution of the remaining immune cells in the graph was statistically significant (P<0.05). They were three types more distributed in the high-risk subgroup. They were macrophage/monocyte, monocyte, and NK cell. And the other five types were more distributed in the low-risk subgroup, which included B cell, endothelial cell, myeloid dendritic cell, neutrophil, and T cell. Fifthly, we wielded QUANTISEQ to draw a diagram based on the Wilcoxon test, which showed the distribution of 11 types of immune cells in the high-risk and low-risk subgroups (**Figure 10H**).

Moreover, we found that the distribution of 7 immune cells in the diagram was statistically significant (P<0.05). And there were two types, including T cell CD4 (non-regulatory) and uncharacterized cells more distributed in the high-risk subgroup. The other five types were more distributed in the low-risk subgroup. They involved B cells, macrophage M2, neutrophils, NK cells, and T cell regulatory (Tregs). Nevertheless, the remaining four types were not statistically significant. They were macrophage M1, monocyte, myeloid dendritic cell, and T cell CD8. Sixthly, we manipulated TIMER to obtain a picture based on the Wilcoxon test, which indicated the distribution of 6 types of immune cells in high-risk and low-risk subgroups (**Figure 10I**).

Furthermore, we found that the distribution of 3 immune cells in the picture was statistically significant (P<0.05). And all the types were more distributed in the low-risk subgroup. They were B cell, myeloid dendritic cell, and T cell CD4. But the rest of the cells were not statistically significant, which included macrophage, neutrophil, and T cell CD8.

Seventhly, we operated TISIDB to draw a diagram based on the Wilcoxon test, which showed the distribution of 28 types of immune cells in the high-risk and low-risk subgroups (**Figure 10J**). In addition, we found that the distribution of 20 immune cells in the graph was statistically significant (P<0.05). And there were seven types more distributed in the high-risk subgroup. They were T cell CD4 central memory, activated CD4 T cell, T cell gamma delta, B cell memory, T cell NK, neutrophil, and T cell CD4 Th2. The other 13 types more distributed in the low-risk subgroup, which included activated B cell, activated dendritic cell, CD56bright natural killer cell, eosinophil, B cell naïve, immature dendritic cell, mast cell, MDSC, monocyte, NK cell, plasmacytoid dendritic cell, T cell follicular helper, type 17 T helper cell. However, the remaining eight types were not statistically significant. There were T cell CD4 activated memory, activated CD8 T cell, T cell CD8 activated memory, CD56dim natural killer cell, T cell CD8 central memory, macrophage, T cell regulatory (Tregs), T cell CD4 Th1. Eighthly, we used XCELL to obtain a picture based on the Wilcoxon test, which indicated the distribution of 36 types of immune cells in the high-risk and low-risk subgroups (**Figure 10K**).

Furthermore, we found that the distribution of 23 immune cells in the picture was statistically significant (P<0.05). And there were seven types more distributed in the high-risk subgroup. They were common lymphoid progenitor, macrophage M1, plasmacytoid dendritic cell, T cell CD4 memory, T cell CD4 Th1, T cell CD4 Th2, T cell CD8 naïve. The other 16 types were more distributed in the low-risk subgroup. They were B cell, CAF, class−switched memory B cell, common myeloid progenitor, endothelial cell, eosinophil, granulocyte−monocyte progenitor, hematopoietic stem cell, macrophage M2, mast cell, myeloid dendritic cell, myeloid dendritic cell activated, T cell CD4 activated memory, T cell CD4 central memory, T cell CD4 naïve, T cell NK. But the remaining 13 types were not statistically significant. They were B cell memory, B cell naïve, B cell plasma, macrophage, monocyte, neutrophil, NK cell, T cell CD4 (non−regulatory), T cell CD8, T cell CD8 central memory, T cell gamma delta, T cell regulatory (Tregs).

Based on Venn diagram, we found that 8 algorithms have multiple overlapping immune cell types (**Figure 10L**). We estimated three types of immune cells threatening the survival of patients in the high-TILPI subgroup based on multiple algorithms (**Table S4**). They were macrophage M0, T cell CD4 Th2, and T cell CD4 memory activated. On the contrary, five immune cells, including B cell, endothelial cell, eosinophil, mast cell, and T cell CD4 memory resting, prolonged the survival (**Table S4**).

**Figure 11:**
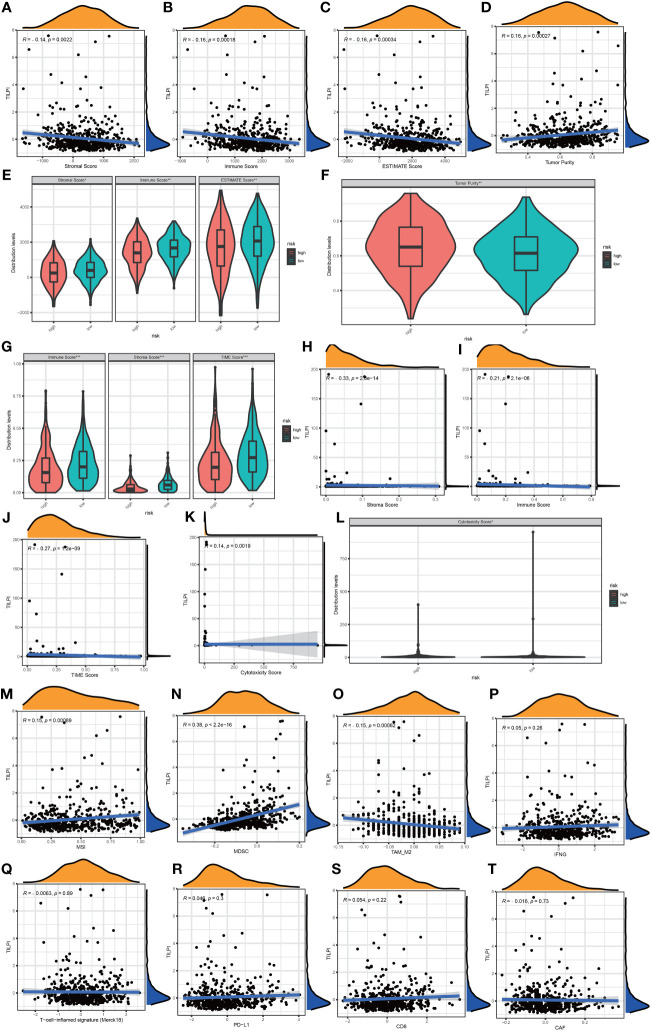
TILPI was negatively correlated with stroma score, immune score, and estimate score and positively related to tumor purity in the ESTIMATE algorithm **(A-F)**. TILPI also was negatively correlated with stroma score, immune score, and TIME score in the XCELL algorithm **(G-J)**. TILPI was positively correlated with the cytotoxicity score of MCPcounter **(K, L)**. MSI and MDSC were positively correlated with TILPI, but the TAM-M2 was the contrary. The other TIME components from TIDE weren’t related to TILPI **(M-T)**. *P < 0.05; **P < 0.01; ***P < 0.001.

**Figure 12:**
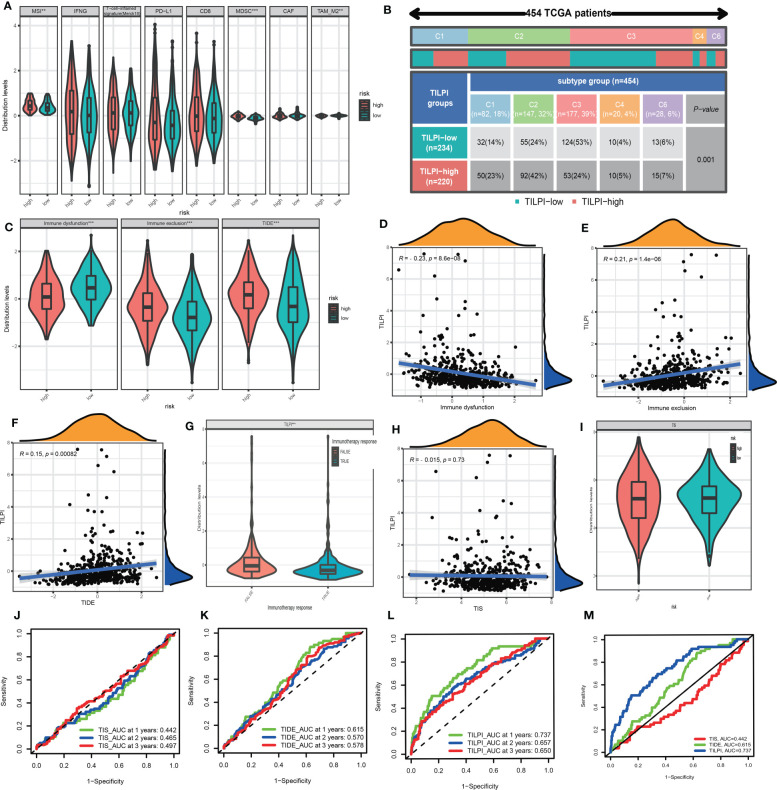
MSI and MDSC were positively correlated with TILPI, but the TAM-M2 was the contrary. The other TIME components from TIDE weren’t related to TILPI **(A)**. TILPI was also correlated with the immune subtype of distribution. The low-TILPI subgroup was more distributed in immune C3 (n=124), and most of the samples in the high-TILPI subgroup were distributed in immune C2 (n=92) **(B)**. The TIDE model proved that the low-TILPI subgroup was more sensitive to immunotherapy **(C-F)**. The correlation between TILPI and immunotherapy once again demonstrated the sensitivity of the low-TILPI subgroup to immunotherapy **(G)**. TILPI wasn’t correlated with the TIS model **(H-I)**. The ROC curves showed TILPI computational framework was better related to prognosis than the TIDE model and TIS model **(J-M)**. **P < 0.01; ***P < 0.001.

TIME has not only immune cells but also numerous stromal components. We obtained the stroma score, immune score, estimate score, and tumor purity from ESTIMATE. Stromal and immune scores were calculated to relate to the levels of stromal invasion and immune cells and thus to infer the tumor tissue’s tumor purity which meant the proportion of tumor cells in TIME. In the same way, stroma score (R=-0.14, P=0.0022), immune score (R=-0.16, P=0.00018), and estimate score (R=-0.16, P=0.00034) were negatively correlated with TILPI (**Figures 11A-C**). But the tumor purity was the opposite. It was positively correlated with TILPI (R=0.16, P=0.00027) (**Figure 11D**). And stroma score, immune score, and estimate score were also higher in the low-TILPI group, while tumor purity was higher in the high-TILPI group (P<0.01) (**Figures 11E, F**). Furthermore, we obtained three kinds of scores (stroma score, immune score, and TIME score) based on XCELL. Stromal score and immune score related to the level of infiltrating stromal and immune cells in TIME. We found that three kinds of scores were all higher in the low-TILPI group (P<0.001). It also verified that three types of scores were all negatively correlated with TILPI (**Figure 11G**). We also analyzed their correlation with TILPI by Pearson correlation analysis, and stroma score (R=-0.33, P<0.001), immune score (R=-0.21, P<0.001), and TIME score (R=-0.27, P<0.001) were all negatively correlated with TILPI (**Figures 11H-J**). Moreover, MCPcounter is a model based on the gene expression matrix, and absolute abundance scores of eight immune cells and two stromal cells were generated for each sample. We first analyzed the Pearson correlation between cytotoxicity score based on MCPcounter and TILPI. According to the results, cytotoxicity score and TILPI were positively correlated (R=0.14, P=0.0019) (**Figure 11K**). To verify this result, we also performed the Wilcoxon test on the TCGA group to observe whether there was a cytotoxicity score difference between the high-TILPI and low-TILPI groups. And the result showed that the cytotoxicity score was higher in the high-TILPI group (P<0.05) (**Figure 11L**), which further verified cytotoxicity score and TILPI were positively correlated. In addition, we got eight types of scores by TIDE: MSI, IFNG, Merck18, PD-L1, CD-8, MDSC, CAF, and TAM-M2. We found that MSI (R=0.15, P=0.00089) and MDSC (R=0.38, P<0.001) were positively correlated with TILPI by Pearson correlation analysis (**Figures 11M, N**). And TAM-M2 was negatively correlated with TILPI (R=-0.15, P=0.00062) (**Figure 11O**). Unfortunately, IFNG, Merck18, PD-L1, CD-8, and CAF were independent of TILPI (P>0.05) (**Figures 11P-T**). In the Wilcoxon test, MSI and MDSC were higher in the high-TILPI group (P<0.01, P<0.001), and TAM-M2 was higher in the low-TILPI group (P<0.01), while IFNG, Merck18, PD-L1, CD-8, and CAF were meaningless (P>0.05) (**Figure 12A**). At last, TILPI was also correlated with the immune subtype of distribution (P = 0.001) (**Figure 12B**). Only 454 samples were corresponding immune subtypes. They were divided into five subtypes, which were immune C1 (n=82), immune C2 (n=147), immune C3 (n=177), immune C4 (n=20), and immune C6 (n=28). The low-TILPI subgroup was more distributed in immune C3 (n=124), and most of the samples in the high-TILPI subgroup were distributed in immune C2 (n=92) (**Figure 12B**).

### 3.5 Association between TILPI and immunotherapy sensitivity

Tumor immune dysfunction and exclusion (TIDE) was a computing framework for evaluating the likelihood of tumor immune escape in gene expression profiles of tumor samples. We calculated the immune dysfunction score, immune exclusion score, and TIDE score. The Wilcoxon test showed immune dysfunction was higher in the low-TILPI group, immune exclusion was higher in the high-TILPI group, and TIDE was higher in the high-TILPI group, which was the most important (**Figure 12C**). The Pearson correlation analysis showed immune dysfunction was negatively correlated with TILPI (R=-0.23, P<0.001) (**Figure 12D**), while immune exclusion (R=0.21, P<0.001) and TIDE (R=0.15, P=0.00082) were positively correlated with TILPI (**Figures 12E, F**). As we know, high TIDE scores indicated severe immune evasion, and it was clear that the high-TILPI group was more prone to immune evasion than the low-TILPI group. Thus, there was no doubt that the low-TILPI group was more suitable for immunotherapy. What’s more, the true immunotherapy response had lower TILPI than the false immunotherapy response (P< 0.001), which further verified that the low-TILPI group was more suitable for immunotherapy in the LUAD patients (**Figure 12G**).

The TIS was a marker of the immune microenvironment gene expression profile. It is based on eighteen genes to relate to the clinical benefit of PD-1-directed therapy. We first analyzed the correlation between TIS and TILPI, but TIS was independent of TILPI (P>0.05) (**Figure 12H**). And the Wilcoxon test showed TIS was no difference between high-TILPI and low-TILPI (**Figure 12I**). Furthermore, we calculated the reliability of three models (TIS, TIDE, TILPI) in LUAD patients’ 1 year, 2 years, and 3 years OS. The results showed that TIS did not do a good job in relating to the OS of LUAD patients (AUC=0.442, AUC=0.465, AUC=0.497) (**Figure 12J**), and TIDE didn’t either (AUC=0.615, AUC=0.570, AUC=0.578) (**Figure 12K**). Fortunately, TILPI had excellent credibility in relating to the OS of LUAD patients (AUC=0.737, AUC=0.657, AUC=0.650) (**Figure 12L**). In summary, TILPI has an advantage over TIS and TIDE, and the time-dependent ROC curves between TILPI (AUC=0.737), TIS (AUC=0.442), and TIDE (AUC=0.615) also verified it (**Figure 12M**).

Immunotherapy associations based on TIDE predictions also apply to LUSC and NSCLC populations. In the LUSC population, TILPI was significantly lower in the immunotherapy-responding group than in the non-responding group (P=0.0015) (**Figure S1H**). This was also the case in NSCLC patients, with the immunotherapy response group having a lower TILPI (P<0.001) (**Figure S1I**). The following five published transcriptomics signatures of immune responses confirmed that the low TILPI group may be more suitable for immunotherapy. As you can see, the low TILPI group had a higher TLS score, implying higher immune activity (**Figures S1J, K**). The low TILPI group induced weaker immune resistance (**Figures S1L, M**) and stronger ability to suppress immune resistance (**Figures S1N, O**), which also indicated that the low TILPI group may be more suitable for immunotherapy. The low TILPI group also had a higher Roh immune score, which was associated with higher immune activation (**Figures S1P, Q**). The Ock anti-CTLA-4 signature expression level of the low TILPI group was higher, which was also associated with better immunotherapy efficacy (**Figures S1R, S**). There was no difference in EaSIeR score between different TILPI groups, but there was a tendency for lower EaSIeR score in the lower TILPI group (**Figures S1T, U**).

### 3.6 Relationship between TILPI and drug sensitivity

**Figure 13:**
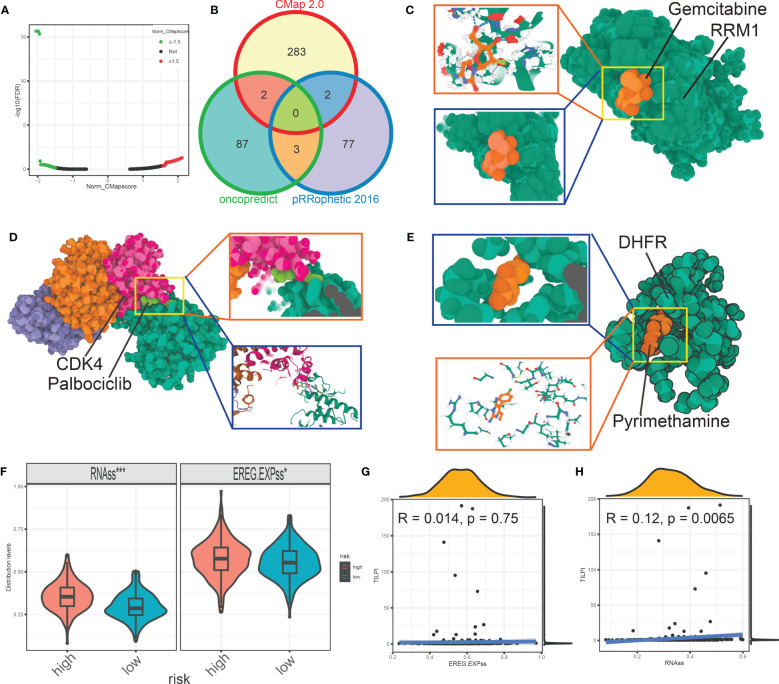
The volcano map showed the qualified drugs based on CMap **(A)**. The intersection analysis estimated seven more reliable drugs based on pRRophetic, oncopredict, and CMap **(B)**. The molecular model of gemcitabine docking RRM1 **(C)**. The molecular model showed the details that palbociclib docking CDK4 **(D)**. Pyrimethamine firmly docked its target protein DHFR **(E)**. The stemness score was different between different TILPI subgroups **(F, G)**. *P < 0.05; ***P < 0.001.

Furthermore, we wished TILPI computing frameworks also relate to sensitive drugs to a specific population. Firstly, we conducted analyses of drug sensitivity based on the pRRophetic package updated in 2016. The judgmental standard of drug sensitivity was IC50. The patients with lower IC50 were sensitive to this drug. The filter was P value of the Wilcoxon test less than 0.05. Then we picked out 12 types of drugs more sensitive in the low-TILPI subgroup, which included AS605240, AZ628, Crizotinib, Erlotinib, KIN001-135, Phenformin, Salubrinal, TAK-715, TL-2-105, WZ3105, YM155, and Z-LLNle-CHO. And 82 types of drugs were also determined for patients with poor prognosis in the high-TILPI subgroup. Secondly, we conducted analyses of drug sensitivity based on the oncopredcit package. The judgmental standard of drug sensitivity was the same as the pRRophetic possessing package. Then 11 types of drugs were identified for the low-TILPI subgroup. They were ABT737, Axitinib, AZD6482, BMS.754807, Doramapimod, GSK269962A, PF.4708671, PRT062607, Ribociclib, SB505124, and ZM447439. And the number of sensitive drugs in the high-TILPI subgroup was 92. Thirdly, we used 95 up-regulated mRNAs from 267 immune TMB-derived oncogenic mRNAs to estimate drugs inhibiting these up-regulated genes base on the Connectivity Map (CMap). Then we got the results of 423,422 lines under different cell lines, dose, and time. We only selected known compounds and targets. And the absolute normalized CMap score of qualified drugs must be greater than 1.5. Therefore, we obtained 285 qualified drugs for LUAD patients (**Figure 13A**).

At last, we intersected the drugs based on pRRophetic, oncopredcit, and CMap for the population with poor prognosis. That is, in the high-TILPI subgroup. In conclusion, we identified seven sensitive drugs: Docetaxel, Gemcitabine, Paclitaxel, Palbociclib, Pyrimethamine, Thapsigargin, and Vinorelbine for poor prognostic population (**Figure 13B**). Docetaxel, Paclitaxel, and Vinorelbine are tubulin inhibitors that target ABCB1, BCL2, CYP2C8, MAP2, MAP4, MAPT, NR1I2, TLR4, and numerous subtypes of TUB proteins. And Gemcitabine is the ribonucleoside reductase inhibitor targeting RRM1, CMPK1, RRM2, and TYMS. Palbociclib is the CDK inhibitor that targets CDK4, CDK6, and CCND3. Pyrimethamine is the dihydrofolate reductase inhibitor and targets DHFRP1, HEXA, STAT3, DHFR, and SLC47A1. Thapsigargin is the ATPase inhibitor that targets ATP2A1.

Furthermore, we constructed molecular docking models to evaluate the affinity of 7 candidate drugs to their targets. Firstly, the best binding energy of Gemcitabine for RRM1 was -66.514 kcal/mol (**Figure 13C**). It indicates that the affinity between Gemcitabine and RRM1 was low, as seen from the figure (**Figure 13C**). Nevertheless, the best binding energy of the docking model between palbociclib and CDK4 was -7.573 kcal/mol (**Figure 13D**). The best binding energy for pyrimethamine to its target DHFR was -4.056 kcal/mol (**Figure 13E**). It was without doubt that palbociclib and pyrimethamine had low binding energy for their targets, indicating high stable binding and potential. Unfortunately, we failed to construct the molecular docking models of docetaxel, thapsigargin, paclitaxel, and vinorelbine. Seven types of sensitive drugs for the high-TILPI subgroup, especially Palbociclib and pyrimethamine, were worth further exploration.

Based on network pharmacology, we next searched for potential targets of candidate drugs targeting LUAD. A total of 8907 LUAD target genes were collected from genecard database. The numbers of drug targets from SwissTargetPrediction, Batmant-TCM, and Pubchem databases were as follows: docetaxel (341 targets), gemcitabine (116 targets), paclitaxel (244 targets), palbociclib (315 targets), pyrimethamine (65 targets), thapsigargin (1337 targets), and vinorelbine (115 targets). There may be 8 targets of docetaxel acting on LUAD, including ABCA3, TP53, STK11, BIRC5, EGFR, ERBB2, KRAS, and RB1 (**Figure S2A**). The potential targets of gemcitabine for LUAD are TP53, KRAS, HYAL2, EGFR, and ERBB2 (**Figure S2B**). The paclitaxel may act on TP53, BIRC5, EGFR, ERBB2, TXNRD1, and KRAS to control the progression of LUAD (**Figure S2C**). For palbociclib, its potential targets for LUAD treatment may be TP53, BIRC5, ERBB2, SMARCA4, MYC, KRAS, BRAF, and RB1(**Figure S2D**). However, the effective target of pyrimethamine for LUAD seems to be only TP53 (**Figure S2E**). The thapsigargin may kill LUAD cells by targeting BIRC5, IRS1, MYC, MVP, HMOX1, ERBB2, CADM1, and TP53 (**Figure S2F**). The vinorelbine may act on SMARCA4, EPCAM, and ERBB2 to treat LUAD (**Figure S2G**). In total, there are 18 possible targets of these 7 drugs for LUAD, including ABCA3, BIRC5, BRAF, CADM1, EGFR, EPCAM, ERBB2, HMOX1, HYAL2, IRS1, KRAS, MVP, MYC, RB1, SMARCA4, STK11, TP53, and TXNRD1 (**Figure S2H**). Of these, the targets BIRC5, ERBB2, KRAS, and TP53 played a role in more than half of the drugs. The R package limma was used to analyze the differences between the high and low TILPI groups, and 57 up-regulated genes were found in the high TILPI group (**Figure S2I**) (**Table S5**). There are 19 up-regulated genes that are potential targets of LUAD, among which ABCC2, F2, GAL, INHBE, and UGT2B7 may be potential targets of docetaxel, paclitaxel, pyrimethamine, and thapsigargin in the treatment of high TILPI group (**Figure S2J**).

In the NCI-60 cell lines of CellMiner database, thirteen of the 57 up-regulated genes of high TILPI group were associated with the therapeutic sensitivity of 7 candidate drugs (P<0.05) (**Table S6**). They are ABCC2, BEST3, CREB3L3, CYP24A1, DSG4, GAL, GIP, IGF2BP1, MUC13, RAB3B, TFF1, TRIM15 and UGT2B7 (**Figure S2K**).

### 3.7 Prediction of tumor evolutionary status based on computing framework

The stemness score reliably evaluates the similarity of tumor cells to stem cells. The higher stemness score was correlated with therapy resistance, tumor biological functions, and clinical characteristics. We found that the high-TILPI subgroup possessed higher EREG-mRNAss and RNA expression-based stemness scores (RNAss) (**Figure 13F**). It could be one of the reasons that the high-TILPI subgroup had a poor prognosis. And TILPI was also positively correlated with EREG-mRNAss but statistically meaningless (R=-0.014, P=0.75) (**Figure 13G**). In addition, TILPI was also positively correlated with RNAss (R=0.12, P=0.0065) (**Figure 13H**).

## 4 Discussion

Lung cancer remained second in the global cancer rankings in 2021 (1–3). And LUAD is the most common histological type of lung cancer (5, 6). So far, traditional imagology and histopathology are still the gold standards for diagnosing and prognosis of LUAD. However, we aimed to construct a prognostic model based on lncRNA expression. Until a few years ago, lncRNA was regarded as a superfluous substance transcribed by genes (49). However, some in-depth studies have proved that lncRNA was involved in the biological activities of genes (50–52). LncRNA is closely related to tumor function. We first identified 267 TMB-derived oncogenic mRNAs and 176 TMB-derived oncogenic lncRNAs, and we obtained 43 immune TMB-derived oncogenic mRNAs and 36 TMB-derived oncogenic lncRNAs based on Pearson analysis. Next, we explored the potential functions of 79 mRNAs and lncRNAs by meta scape. And then, we further found the pathogenesis pathways of LUAD. We ascertained 30 GO pathways and 21 KEGG pathways in functional enrichment analysis.

Based on KM method analyses, univariate Cox proportional risk regression, and multivariate Cox proportional risk regression, we further study found that six lncRNAs in TILncSet expression level were closely related to the patient’s OS, and all of them were a negative correlation, in other words, they were risk factors (AC091057.1, AC129492.1, AC112721.1, TARID, AC114763.1, LINC00592). Some scholars also have pointed out that AC091057.1 is a risk factor for LUAD patients (53). Other researchers have found that AC129492.1 impacts the prognosis of patients with hepatocellular carcinoma, colon cancer, and osteosarcoma (54–56). It has been reported that AC112721.1 is abnormally expressed in patients with breast cancer and bladder cancer (57, 58). Confusingly, TARID has been shown to activate the expression of the tumor suppressor gene TCF21 by inducing promoter demethylation (59, 60), which is contrary to our results, and the specific reasons remain to be further studied. LINC00592 is ferroptosis-related lncRNA, which has been identified as an independent prognostic predictor of LUAD and may be involved in the immune response to LUAD (61). It can also be used as a prognostic marker for disease-free survival in patients with gastric cancer (62) and is differentially expressed in cervical cancer (63). Unfortunately, no studies on AC114763.1 have yet to be researched. It is expected that these lncRNAs will be further studied.

Based on TMB, we built a novel computing framework called TILPI. It was an innovative step forward. TILPI calculated a risk score for each patient based on the expression of TILncSet. After testing, TILPI distinguished the prognosis of different risk score subgroups well in all groups (training group, test group, and TCGA group), and the higher TILPI, the worse prognosis. The reliability of TILPI was verified, as the AUC value of the TILPI based on time-dependent ROC curves in all groups was higher than or equal to 0.73.

Furthermore, the independence and correlation analysis of some clinical factors were carried out, and the results showed that TILPI was closely related to the degree of smoking, pathological T, pathological N, and pathological stage. Studies have found that, compared with the smoker LUADs, never-smoker LUADs have a higher prevalence of clinically actionable driver alterations (78%-92% v 49.5%; P<.0001) (64). It also suggests that never-smoking patients have a better prognosis. And TILPI wasn’t associated with and independent of race and age. However, studies have found that East Asian LUADs have more stable genomes and better prediction accuracy than European LUADs (65). It may be due to the improper grouping method and insufficient sample size, which made for our failure to find the relationship between race and TILPI, and further improvement is needed. The specific relationship and mechanism between TILPI and other clinical factors remain to be further studied. Fortunately, the Norman plot based on TILPI could still relate to the prognosis of LUAD patients, and we look forward to its clinical performance in the future. Yan Li et al. presented a framework called bioRFR to quantify wellness-to-disease transition in cancer patients by gene expression. They considered that cancer does not progress linearly, making it difficult or impossible to recover once it passes a tipping point. BioRFR was able to identify if a patient has passed this tipping point and provide personalized treatment. We must consider it in the future (66).

We also found ten pathways pathogenic to LUAD and six protectives to LUAD patients through gene enrichment. The prognosis of LUAD patients could be improved by inhibiting these pathogenic pathways or activating these protective pathways. It was a significant finding. Hopefully, it will be validated in the clinic. In summary, we have constructed a new and effective prognostic model for LUAD, accurately distinguishing between low-risk and high-risk LUAD patients. Compared with expensive molecular tests, TILPI is cheaper and more convenient. However, our research is still limited to calculation and analysis, and biological studies are required in the future.

Then, we used eight algorithms to describe the immune infiltration landscape, and we screened out meaningful immune cells by the Wilcoxon test, which were selected from eight immune infiltration landscape sets. Then we took the intersection. Then we ended up with eight immune cells. They were B cell, endothelial cell, eosinophil, mast cell, T cell CD4 memory resting, T cell CD4 Th2, macrophage M0, and T cell CD4 memory activated. B cell, endothelial cell, eosinophil, mast cell, and T cell CD4 memory resting was more in the low-risk subgroup. T cell CD4 Th2, macrophage M0, and T cell CD4 memory activated were more in the high-risk subgroup. We also used ESTIMATE and XCELL algorithms to calculate the stroma score of each sample and combined them with the immune score to reflect tumor purity. The two algorithms’ stroma scores were negatively correlated with TILPI. The higher the stroma score was, the lower TILPI was. As predicted, tumor purity was higher in the high TILPI group.

The predicted immunotherapy response based on TIDE suggested that LUAD patients with low TILPI may be better candidates for immunotherapy, and this possibility applied to LUSC and NSCLC patients as well. The five published transcriptomics signatures of immune responses confirmed that the low TILPI group may be more suitable for immunotherapy. They were the TLS signature, Jerby-Arnon immune resistance program, Roh immune score, Ock anti-CTLA-4 signature, and EaSIeR model. In conclusion, TILPI is a good predictor of TME status and is superior to TIDE and TIS. In the study of Xu et al., TIDE was also used to relate to the effect of immunotherapy in patients. The difference is that their results showed that patients in the high-risk group responded better to immunotherapy, while we concluded that the low-risk group responded better to immunotherapy (67). Further research is required on this issue.

We also reviewed the latest developments of seven sensitive drugs. Network pharmacological analysis suggested that there were 18 potential therapeutic targets for LUAD, including ABCA3, BIRC5, BRAF, CADM1, EGFR, EPCAM, ERBB2, HMOX1, HYAL2, IRS1, KRAS, MVP, MYC, RB1, SMARCA4, STK11, TP53, and TXNRD1. Differential analysis based on R package limma found 57 genes up-regulated in the high TILPI group. Among them, ABCC2, F2, GAL, INHBE, and UGT2B7 may be potential targets of docetaxel, paclitaxel, pyrimethamine, and thapsigargin in the treatment of high TILPI group. In the NCI-60 cell lines of CellMiner database, thirteen of the 57 up-regulated genes of high TILPI group were associated with the therapeutic sensitivity of 7 candidate drugs. Both docetaxel and paclitaxel belonged to taxanes, which, combined with platinum drugs, was the first-line treatment option for LUAD. It was reported that docetaxel prolonged OS versus ICI in NSCLC patients (68). Other researchers have also combined paclitaxel with other drugs, such as nanoparticle albumin and ICI (69, 70). Furthermore, gemcitabine plus platinum was the standard chemotherapy for squamous NSCLC. And some novel research proved that the combination of gemcitabine and other drugs exhibited synergistic antitumor efficacy, which included albumin-bound paclitaxel and ICI (71, 72). In addition, vinorelbine was not a new chemotherapy drug, but it still has powerful effects (73, 74). As for CDK inhibitors, palbociclib was more commonly used in breast cancer patients with RB mutations but has recently been studied in NSCLC (75, 76). Pyrimethamine is an antimalarial drug and has also been proven to have antitumor activity (77). But the clinical research between pyrimethamine and chemotherapy for lung cancer was lacking. This clinical research absence also existed in thapsigargin. In conclusion, the dominant position of docetaxel, paclitaxel, gemcitabine, and vinorelbine in the chemotherapy of LUAD must be emphasized. Nevertheless, the potential significance of palbociclib, pyrimethamine, and thapsigargin in the chemotherapy of LUAD waited for further research.

The computing framework combined with prognosis may be novel research related to immunotherapy and chemotherapy. This computing framework also played some roles but also possessed limitations. Firstly, the details of TILncSig affecting OS were waiting for deeper exploration by biologists. Secondly, the details of the immune cells differently distributed in different TILPI subgroups that affected OS need further biological research. Thirdly, we believed that the low-TILPI subgroup was more sensitive to immunotherapy, but this conclusion needs to be verified in the clinic. Fourthly, the effects of 7 sensitive drugs in the high-TILPI subgroup must also be researched in the clinic. Fifthly, they need more abundant samples. The external datasets should be employed for validation rather than our limited TCGA datasets. Sixthly, prospective analyses of the TILPI computing framework are required because all datasets in the study are retrospective. In any case, we will try our best to solve these problems step by step in further research.

## 5 Conclusion

We identified TILncSig based on TMB-related genes by WGCNA, oncogenes, and immune genes in LUAD. Then we construct the TILPI computing framework to relate to individual prognosis. TILPI could also map the immune infiltration landscape of immune cells, tumor cells, and stromal components based on creatively combined analyses of multiple algorithms. Furthermore, the TILPI computing framework successfully identified different prognostic LUAD populations and selected sensitive immunotherapy/chemotherapy for them. We believed that the low-TILPI subgroup was more sensitive to ICI, and the high-TILPI subgroup had a better effect on seven drugs.

## Data availability statement

The original contributions presented in the study are included in the article/**Supplementary Material**. Further inquiries can be directed to the corresponding author.

## Ethics statement

The studies involving human participants were reviewed and approved by Guangdong Medical University ethics committee. The patients/participants provided their written informed consent to participate in this study.

## Author contributions

XZ conceived the work. CW and WZ wrote and drafted the manuscript. CW, WZ, FH, WH, and XX discussed and edited the manuscript. XZ checked the accuracy of statistics and bioinformatics as an expert in statistics and bioinformatics. All authors contributed to the article and approved the submitted version.

## Supplementary material

Supplementary Figure 1 AC114763.1, LINC00592, TARID, and AC091057.1 independently predicted patient survival **(A-D)**. AC129492.1 and AC112721.1, on the other hand, did not perform well **(E, F)**. The survival probability of NSCLC patients in the high TILPI group was significantly lower than those in the low TILPI group **(G)**. In the LUSC population, TILPI was significantly lower in the immunotherapy-responding group than in the non-responding group **(H)**. Patients with NSCLC who responded to immunotherapy had a lower TILPI **(I)**. The low TILPI group had a higher TLS score **(J, K)**. The low TILPI group induced weaker immune resistance **(L, M)** and stronger ability to suppress immune resistance **(N, O)**. The low TILPI group also had a higher Roh immune score **(P, Q)**. The Ock anti-CTLA-4 signature expression level of the low TILPI group was higher **(R, S)**. There was no difference in EaSIeR score between different TILPI groups, **(T, U)**.

Supplementary Figure 2 There may be 8 targets of docetaxel acting on LUAD **(A)**. The potential targets of gemcitabine for LUAD are TP53, KRAS, HYAL2, EGFR, and ERBB2 **(B)**. The paclitaxel may act on TP53, BIRC5, EGFR, ERBB2, TXNRD1, and KRAS to control the progression of LUAD **(C)**. For palbociclib, there may be as many as eight targets for LUAD **(D)**. The effective target of pyrimethamine for LUAD seems to be only TP53 **(E)**. The thapsigargin may kill LUAD cells by eight targets **(F)**. The vinorelbine may act on SMARCA4, EPCAM, and ERBB2 to treat LUAD **(G)**. In total, there are 18 possible targets of these 7 drugs for LUAD **(H)**. Based on differential expression analysis, 57 up-regulated genes were found in the high TILPI group **(I)**. There are 19 up-regulated genes that are potential targets of LUAD **(J)**. In the NCI-60 cell lines of CellMiner database, thirteen of the 57 up-regulated genes of high TILPI group were associated with the therapeutic sensitivity of 7 candidate drugs **(K)**.

## Glossary

**Table:**

3D PCA	3D principal component analysis
AUC	area under curve
BP	biological process
CAF	cancer associated fibroblast
CAR10	CAR intergenic 10
CC	cell component
CIBERSORT	Cell type Identification by Estimating Relative Subsets of RNA Transcripts
CIBERSORT-ABS	CIBERSORT-absolute mode
CMap	connectivity map
CTL	cytotoxic T lymphocyte
EGFR	epidermal growth factor
EPIC	Estimating the Proportions of Immune and Cancer cells
EREG-mRNAss	epigenetically regulated-mRNA expression-based stemness score
ESTIMATE	Estimation of Stromal and Immune cells in Malignant Tumor tissues using Expression data
FDR	false discovery rate
GEO	Gene Expression Omnibus
GO	Gene Ontology
GS	gene significance
GSEA	gene set enrichment analysis
GTEx	Genotype-Tissue Expression
IC50	semi-inhibitory concentration
ICI	immune checkpoint inhibitors
IFNG	interferon gamma
ImmPort	immunology database and analysis portal
InnateDB	systems biology of the innate immune response
KEGG	Kyoto Encyclopedia of Genes and Genomes
KM	Kaplan-Meier
lncRNA	long non-coding RNA
LUAD	lung adenocarcinoma
LUSC	lung squamous cell carcinomas
M	distant metastasis
MCODE	Molecular Complex Detection
MCPCOUNTER	Microenvironment Cell Populations-counter
MDSC	myeloid-derived suppressor cell
ME	module eigengene
MF	molecular function
MM	module membership
MSI	microsatellite instability
N	regional lymph nodes
NSCLC	non-small cell lung cancer
OS	overall survival
PPI	protein-protein interaction
QUANTISEQ	Quantifying Immune Contexture of Human Tumors
RNAss	RNA expression-based stemness score
ROC	receiver operating characteristic
T	primary tumor
TCGA	The Cancer Genome Atlas
TIDE	tumor immune dysfunction and exclusion
TIgeneSet	TMB-derived immune gene set
TILncSet	TMB-derived immune lncRNA set
TILncSig	TMB-derived immune lncRNA signature
TILPI	TMB-derived immune lncRNA prognostic index
TIME	Tumor immune microenvironment
TIMER	Tumor Immune Estimation Resource
TImSet	TMB-derived immune mRNA set
TIS	tumor inflammation signature
TISIDB	Tumor and Immune System Interaction Database
TLS	tertiary lymphoid structures signature
TMB	tumor mutation burden
TOM	Topological Overlap Matrix
TPM	transcripts per kilobase of exon model per million mapped reads
Tregs	T cell regulatory
TRRUST	transcriptional regulatory relationships unraveled by sentence-based text mining
WGCNA	weighted gene co-expression network analysis
XCELL	digitally portraying the tissue cellular heterogeneity landscape
YB-1	Y-box-binding protein 1.
