# Supplementary figures and images for: A tumor mutational burden-derived immune computational framework selects sensitive immunotherapy/chemotherapy for lung adenocarcinoma populations with different prognoses
Source: Front Oncol. 2023 Jun 30;13:1104137. doi: 10.3389/fonc.2023.1104137 (PMC10349266; doi:10.3389/fonc.2023.1104137)

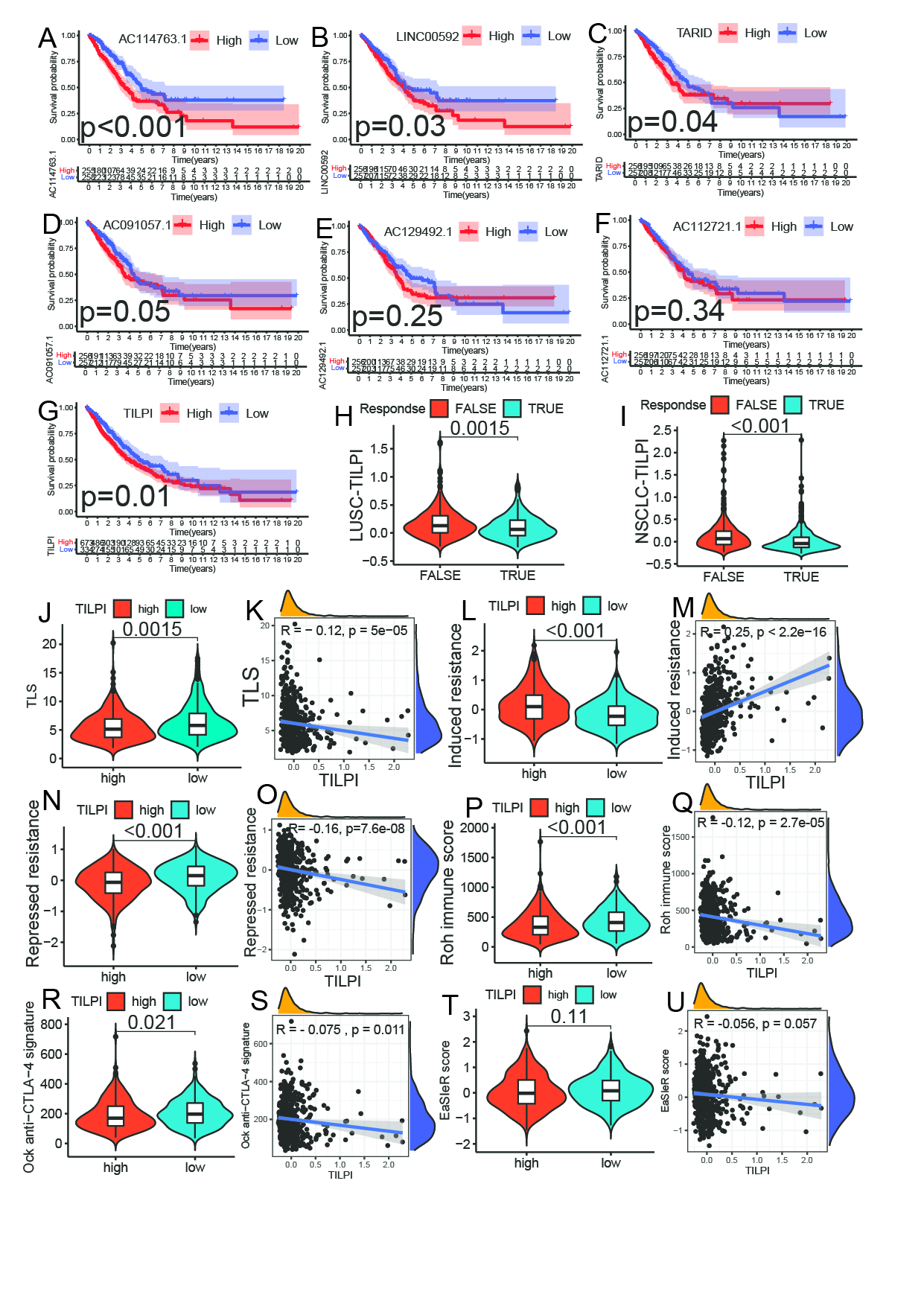

Supplement: Supplementary Figure 1 — AC114763.1, LINC00592, TARID, and AC091057.1 independently predicted patient survival (A-D). AC129492.1 and AC112721.1, on the other hand, did not perform well (E, F). The survival probability of NSCLC patients in the high TILPI group was significantly lower than those in the low TILPI group (G). In the LUSC population, TILPI was significantly lower in the immunotherapy-responding group than in the non-responding group (H). Patients with NSCLC who responded to immunotherapy had a lower TILPI (I). The low TILPI group had a higher TLS score (J, K). The low TILPI group induced weaker immune resistance (L, M) and stronger ability to suppress immune resistance (N, O). The low TILPI group also had a higher Roh immune score (P, Q). The Ock anti-CTLA-4 signature expression level of the low TILPI group was higher (R, S). There was no difference in EaSIeR score between different TILPI groups, (T, U). [file Image_1.tif]

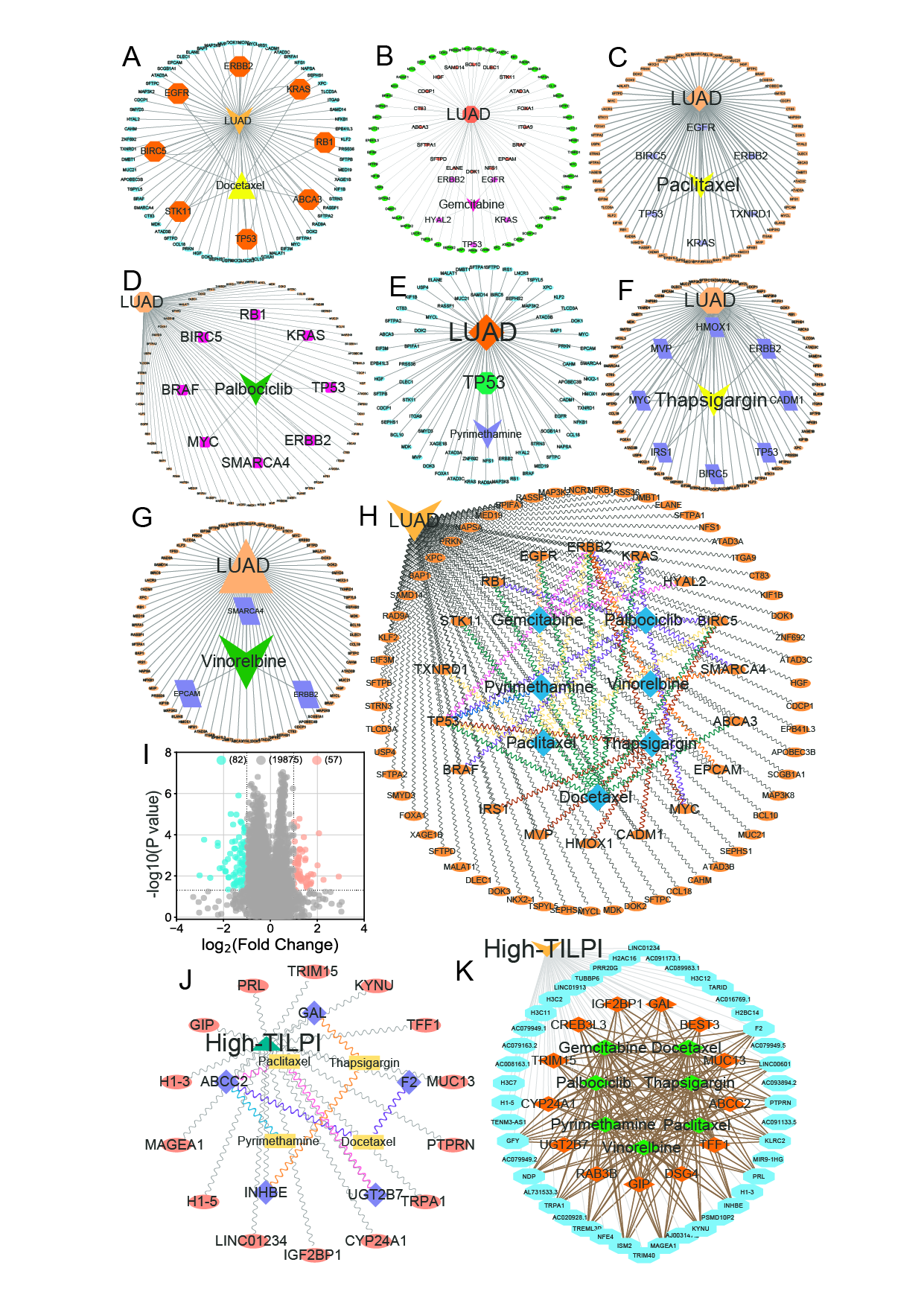

Supplement: Supplementary Figure 2 — There may be 8 targets of docetaxel acting on LUAD (A). The potential targets of gemcitabine for LUAD are TP53, KRAS, HYAL2, EGFR, and ERBB2 (B). The paclitaxel may act on TP53, BIRC5, EGFR, ERBB2, TXNRD1, and KRAS to control the progression of LUAD (C). For palbociclib, there may be as many as eight targets for LUAD (D). The effective target of pyrimethamine for LUAD seems to be only TP53 (E). The thapsigargin may kill LUAD cells by eight targets (F). The vinorelbine may act on SMARCA4, EPCAM, and ERBB2 to treat LUAD (G). In total, there are 18 possible targets of these 7 drugs for LUAD (H). Based on differential expression analysis, 57 up-regulated genes were found in the high TILPI group (I). There are 19 up-regulated genes that are potential targets of LUAD (J). In the NCI-60 cell lines of CellMiner database, thirteen of the 57 up-regulated genes of high TILPI group were associated with the therapeutic sensitivity of 7 candidate drugs (K). [file Image_2.tif]
