# Supplementary material for: A tumor mutational burden-derived immune computational framework selects sensitive immunotherapy/chemotherapy for lung adenocarcinoma populations with different prognoses
Source: Front Oncol. 2023 Jun 30;13:1104137. doi: 10.3389/fonc.2023.1104137 (PMC10349266; doi:10.3389/fonc.2023.1104137)
Supplement: Supplementary file 3 [file Table_1.docx]

**Table S1.** Statistics of clinical characteristics for three groups.

| **Item** | **Type** | **The training group** | **The testing group** | **The TCGA group** | **P value** |
| --- | --- | --- | --- | --- | --- |
| **Survival status** | Dead | 88(34.24%) | 97(37.89%) | 185(36.06%) | 0.4421 |
|  | Alive | 169(65.76%) | 159(62.11%) | 328(63.94%) |  |
| **OS (years)** | [0,1) | 59(22.96%) | 51(19.92%) | 110(21.44%) | 0.5103 |
|  | [1,5) | 173(67.32%) | 171(66.8%) | 344(67.06%) |  |
|  | [5,10) | 22(8.56%) | 28(10.94%) | 50(9.75%) |  |
|  | >=10 | 3(1.17%) | 6(2.34%) | 9(1.75%) |  |
| **Age** | <=65 | 127(49.42%) | 130(50.78%) | 257(50.1%) | 0.8252 |
|  | >65 | 130(50.58%) | 126(49.22%) | 256(49.9%) |  |
| **Gender** | FEMALE | 141(54.86%) | 136(53.12%) | 277(54%) | 0.7592 |
|  | MALE | 116(45.14%) | 120(46.88%) | 236(46%) |  |
| **Race** | WHITE | 199(77.43%) | 204(79.69%) | 403(78.56%) | 0.5258 |
|  | ASIAN | 6(2.33%) | 2(0.78%) | 8(1.56%) |  |
|  | BLACK OR AFRICAN AMERICAN | 35(13.62%) | 32(12.5%) | 67(13.06%) |  |
|  | AMERICAN INDIAN OR ALASKA NATIVE | 0(0%) | 1(0.39%) | 1(0.19%) |  |
|  | unknown | 17(6.61%) | 17(6.64%) | 34(6.63%) |  |
| **Smoking** | 1 | 45(17.51%) | 31(12.11%) | 76(14.81%) | 0.2708 |
|  | 2 | 54(21.01%) | 66(25.78%) | 120(23.39%) |  |
|  | 3 | 65(25.29%) | 70(27.34%) | 135(26.32%) |  |
|  | 4 | 92(35.8%) | 86(33.59%) | 178(34.7%) |  |
|  | 5 | 1(0.39%) | 3(1.17%) | 4(0.78%) |  |
| **T** | T1 | 82(31.91%) | 86(33.59%) | 168(32.75%) | 0.8942 |
|  | T2 | 139(54.09%) | 139(54.3%) | 278(54.19%) |  |
|  | T3 | 24(9.34%) | 21(8.2%) | 45(8.77%) |  |
|  | T4 | 11(4.28%) | 8(3.12%) | 19(3.7%) |  |
|  | TX | 1(0.39%) | 2(0.78%) | 3(0.58%) |  |
| **N** | N0 | 162(63.04%) | 167(65.23%) | 329(64.13%) | 0.5694 |
|  | N1 | 47(18.29%) | 50(19.53%) | 97(18.91%) |  |
|  | N2 | 43(16.73%) | 31(12.11%) | 74(14.42%) |  |
|  | N3 | 1(0.39%) | 1(0.39%) | 2(0.39%) |  |
|  | NX | 4(1.56%) | 7(2.73%) | 11(2.14%) |  |
| **M** | M0 | 163(63.42%) | 185(72.27%) | 348(67.84%) | 0.1002 |
|  | M1 | 15(5.84%) | 11(4.3%) | 26(5.07%) |  |
|  | MX | 79(30.74%) | 60(23.44%) | 139(27.1%) |  |
| **Stage** | Stage I | 135(52.53%) | 142(55.47%) | 277(54%) | 0.7758 |
|  | Stage II | 61(23.74%) | 63(24.61%) | 124(24.17%) |  |
|  | Stage III | 47(18.29%) | 39(15.23%) | 86(16.76%) |  |
|  | Stage IV | 14(5.45%) | 12(4.69%) | 26(5.07%) |  |
