# Supplementary material for: A tumor mutational burden-derived immune computational framework selects sensitive immunotherapy/chemotherapy for lung adenocarcinoma populations with different prognoses
Source: Front Oncol. 2023 Jun 30;13:1104137. doi: 10.3389/fonc.2023.1104137 (PMC10349266; doi:10.3389/fonc.2023.1104137)
Supplement: Supplementary file 4 [file Table_2.docx]

**Table S2.** Independence analyses of clinical characteristics and TILPI in three groups.

|  |  | **Indep-uniCox** | | | | **Indep-multiCox** | | | |
| --- | --- | --- | --- | --- | --- | --- | --- | --- | --- |
|  |  | HR | HR.95L | HR.95H | P value | HR | HR.95L | HR.95H | P value |
| **The training group** | **age** | 1.0277 | 1.0060 | 1.0498 | 0.0121 | - | - | - | - |
|  | **gender** | - | - | - | - | - | - | - | - |
|  | **smoking** | - | - | - | - | - | - | - | - |
|  | **T** | 1.5006 | 1.1509 | 1.9565 | 0.0027 | - | - | - | - |
|  | **N** | 1.6760 | 1.3137 | 2.1383 | 0.0000 | 1.6249 | 1.1093 | 2.3803 | 0.0127 |
|  | **M** | - | - | - | - | - | - | - | - |
|  | **stage** | 1.4959 | 1.2286 | 1.8214 | 0.0001 | - | - | - | - |
|  | **TILPI** | 1.1874 | 1.1223 | 1.2563 | 0.0001 | 1.1714 | 1.0978 | 1.2500 | 0.0001 |
| **The testing group** | **age** | 1.0059 | 0.9887 | 1.0234 | 0.5060 | - | - | - | - |
|  | **gender** | 1.1812 | 0.7920 | 1.7616 | 0.4142 | - | - | - | - |
|  | **smoking** | 1.0380 | 0.8605 | 1.2522 | 0.6966 | - | - | - | - |
|  | **T** | 1.5226 | 1.1663 | 1.9876 | 0.0020 | 1.0627 | 0.7657 | 1.4751 | 0.7161 |
|  | **N** | 1.6376 | 1.2895 | 2.0796 | 0.0001 | 0.9152 | 0.5495 | 1.5244 | 0.7336 |
|  | **M** | 3.8615 | 1.8968 | 7.8612 | 0.0002 | 0.7047 | 0.1650 | 3.0095 | 0.6366 |
|  | **stage** | 1.8696 | 1.5281 | 2.2875 | 0.0000 | 1.9103 | 1.0978 | 3.3239 | 0.0220 |
|  | **TILPI** | 1.0167 | 1.0088 | 1.0247 | 0.0000 | 1.0168 | 1.0083 | 1.0254 | 0.0001 |
| **The TCGA group** | **age** | 1.0152 | 1.0017 | 1.0288 | 0.0274 | 1.0101 | 0.9931 | 1.0275 | 0.2445 |
|  | **gender** | 1.1024 | 0.8255 | 1.4721 | 0.5089 | - | - | - | - |
|  | **smoking** | 1.0441 | 0.9127 | 1.1945 | 0.5295 | - | - | - | - |
|  | **T** | 1.5136 | 1.2550 | 1.8255 | 0.0000 | 1.2409 | 0.9703 | 1.5869 | 0.0855 |
|  | **N** | 1.6558 | 1.3981 | 1.9610 | 0.0000 | 1.3963 | 0.9685 | 2.0131 | 0.0737 |
|  | **M** | 2.1503 | 1.2557 | 3.6823 | 0.0053 | 1.1370 | 0.4258 | 3.0358 | 0.7978 |
|  | **stage** | 1.6489 | 1.4350 | 1.8948 | 0.0000 | 1.2157 | 0.8120 | 1.8199 | 0.3428 |
|  | **TILPI** | 1.0149 | 1.0079 | 1.0219 | 0.0000 | 1.0143 | 1.0042 | 1.0244 | 0.0053 |
