# Supplementary material for: A tumor mutational burden-derived immune computational framework selects sensitive immunotherapy/chemotherapy for lung adenocarcinoma populations with different prognoses
Source: Front Oncol. 2023 Jun 30;13:1104137. doi: 10.3389/fonc.2023.1104137 (PMC10349266; doi:10.3389/fonc.2023.1104137)
Supplement: Supplementary file 5 [file Table_3.docx]

**Table S3.** Clinical characteristics independence and relationship analysis of TILPI.

|  |  | **Clinical characteristics independence analysis** | | | | |  | **Clinical characteristics correlation analysis** | | | | | **Conclusion** |
| --- | --- | --- | --- | --- | --- | --- | --- | --- | --- | --- | --- | --- | --- |
| **Item** | **Type** | **TILPI-high** | **TILPI-low** | **Total** | **P value** | **Statistical meaning** | **Type** | **TILPI-low** | **TILPI-high** | **Total** | **P value** | **Statistical meaning** |  |
| **Age** | **<=65** | 137 | 120 | 257 | 0.057 | × | **<=65** | 120 | 137 | 257 | 0.402 | × | - |
|  | **>65** | 126 | 130 | 256 | <0.001 |  | **>65** | 130 | 126 | 256 |  |  |  |
| **Gender** | **FEMALE** | 119 | 158 | 277 | <0.001 | √ | **FEMALE** | 24 | 52 | 76 | 0.001 | √ | - |
|  | **MALE** | 144 | 92 | 236 | 0.004 |  | **MALE** | 119 | 92 | 211 |  |  |  |
| **Smoking** | **1** | 25 | 51 | 76 | 0.01 | × | **1** | 51 | 25 | 76 | 0.001 | √ | Relevantly |
|  | **2** | 78 | 42 | 120 | 0.998 |  | **2** | 42 | 78 | 120 |  |  |  |
|  | **3** | 60 | 75 | 135 | 0.036 |  | **3** | 75 | 60 | 135 |  |  |  |
|  | **4** | 97 | 81 | 178 | <0.001 |  | **4** | 81 | 97 | 178 |  |  |  |
|  | **5** | 3 | 1 | 4 | 1 |  | **5** | 1 | 3 | 4 |  |  |  |
| **Race** | **AMERICAN INDIAN OR ALASKA NATIVE** | 0 | 1 | 1 | - | × | **AMERICAN INDIAN OR ALASKA NATIVE** | 1 | 0 | 1 | 0.208 | × | - |
|  | **ASIAN** | 3 | 7 | 10 | 0.527 |  | **ASIAN** | 7 | 3 | 10 |  |  |  |
|  | **BLACK OR AFRICAN AMERICAN** | 29 | 38 | 67 | 0.369 |  | **BLACK OR AFRICAN AMERICAN** | 38 | 29 | 67 |  |  |  |
|  | **WHILT** | 209 | 194 | 403 | <0.001 |  | **WHILT** | 194 | 209 | 403 |  |  |  |
|  | **unknown** | 22 | 12 | 34 | 0.231 |  | **unknown** | 12 | 22 | 34 |  |  |  |
| **T** | **T1** | 62 | 106 | 168 | 0.386 | × | **T1** | 106 | 62 | 168 | 0.001 | √ | Relevantly |
|  | **T2** | 158 | 120 | 278 | 0.007 |  | **T2** | 120 | 158 | 278 |  |  |  |
|  | **T3** | 30 | 15 | 45 | 0.001 |  | **T3** | 15 | 30 | 45 |  |  |  |
|  | **T4** | 12 | 7 | 19 | 0.031 |  | **T4** | 7 | 12 | 19 |  |  |  |
| **N** | **N0** | 155 | 174 | 329 | 0.003 | × | **N0** | 174 | 155 | 329 | 0.004 | √ | Relevantly |
|  | **N1** | 55 | 42 | 97 | 0.309 |  | **N1** | 42 | 55 | 97 |  |  |  |
|  | **N2** | 50 | 24 | 74 | 0.026 |  | **N2** | 24 | 50 | 74 |  |  |  |
|  | **N3** | 0 | 2 | 2 | - |  | **N3** | 2 | 0 | 2 |  |  |  |
| **M** | **M0** | 187 | 161 | 348 | <0.001 | × | **M0** | 161 | 187 | 348 | 0.344 | × | - |
|  | **M1** | 17 | 9 | 26 | 0.634 |  | **M1** | 9 | 17 | 26 |  |  |  |
| **Stage** | **Stage I** | 118 | 159 | 277 | 0.106 | × | **Stage I** | 159 | 118 | 277 | 0.001 | √ | Relevantly |
|  | **Stage II** | 73 | 51 | 124 | 0.046 |  | **Stage II** | 51 | 73 | 124 |  |  |  |
|  | **Stage III** | 55 | 31 | 86 | 0.007 |  | **Stage III** | 31 | 55 | 86 |  |  |  |
|  | **Stage IV** | 17 | 9 | 26 | 0.886 |  | **Stage IV** | 9 | 17 | 26 |  |  |  |
