# Supplementary material for: A tumor mutational burden-derived immune computational framework selects sensitive immunotherapy/chemotherapy for lung adenocarcinoma populations with different prognoses
Source: Front Oncol. 2023 Jun 30;13:1104137. doi: 10.3389/fonc.2023.1104137 (PMC10349266; doi:10.3389/fonc.2023.1104137)
Supplement: Supplementary file 6 [file Table_4.docx]

**Table S4.** Mapping of immune landscape based on computational framework.

|  | **More distributed in low-risk subgroup** | **More distributed in high-risk subgroup** | **Meaninglessly** |
| --- | --- | --- | --- |
| **CIBERSORT** | B cell memory, dendritic cell resting, monocyte, mast cell resting, eosinophil, T cell CD4 memory resting, T cell regulatory (Tregs) | macrophage M0, macrophage M1, plasma cell, T cell CD4 memory activated | B cell naive, dendritic cell activated, macrophage M2, mast cell activated, neutrophil, NK cell activated, NK cell resting, T cell CD4 naïve, T cell CD8, T cell follicular helper, T cell gamma delta |
|  |  |  |  |
|  |  |  |  |
|  |  |  |  |
|  |  |  |  |
|  |  |  |  |
|  |  |  |  |
| **CIBERSORT-ABS** | B cell memory, eosinophil, macrophage M2, mast cell activated, monocyte, myeloid dendritic cell resting, T cell CD4 memory resting, T cell regulatory (Tregs) | macrophage M0, mast cell resting, T cell CD4 memory activated | B cell naive, B cell plasma, macrophage M1, myeloid dendritic cell activated, neutrophil, NK cell activated, NK cell resting, T cell CD4 naive, T cell CD8, T cell follicular helper. |
|  |  |  |  |
|  |  |  |  |
|  |  |  |  |
|  |  |  |  |
|  |  |  |  |
| **EPIC** | B cell, endothelial cell | NK cell | CAF, macrophage, T cell CD4, T cell CD8, uncharacterized cell |
|  |  |  |  |
|  |  |  |  |
| **MCPCOUNTER** | B cell, endothelial cell, myeloid dendritic cell, neutrophil, T cell | macrophage/monocyte, monocyte, NK cell | CAF, T cell CD8 |
|  |  |  |  |
| **QUANTISEQ** | B cell, macrophage M2, neutrophil, NK cell, T cell regulatory (Tregs) | T cell CD4 (non-regulatory), uncharacterized cell | macrophage M1, monocyte, myeloid dendritic cell, T cell CD8 |
|  |  |  |  |
| **TIMER** | B cell, myeloid dendritic cell, T cell CD4 | - | macrophage, neutrophil, T cell CD8 |
|  |  |  |  |
| **TISIDB** | activated B cell, activated dendritic cell, CD56bright natural killer cell, eosinophil, B cell naïve, immature dendritic cell, mast cell, MDSC, monocyte, NK cell, plasmacytoid dendritic cell, T cell follicular helper, type 17 T helper cell | T cell CD4 central memory, activated CD4 T cell, T cell gamma delta, B cell memory, T cell NK, neutrophil, T cell CD4 Th2 | T cell CD4 activated memory, activated CD8 T cell, T cell CD8 activated memory, CD56dim natural killer cell, T cell CD8 central memory, macrophage, T cell regulatory (Tregs), T cell CD4 Th1 |
|  |  |  |  |
|  |  |  |  |
|  |  |  |  |
|  |  |  |  |
| **XCELL** | B cell, CAF, class−switched memory B cell, common myeloid progenitor, endothelial cell, eosinophil, granulocyte−monocyte progenitor, hematopoietic stem cell, macrophage M2, mast cell, myeloid dendritic cell, myeloid dendritic cell activated, T cell CD4 activated memory, T cell CD4 central memory, T cell CD4 naïve, T cell NK | Common lymphoid progenitor, macrophage M1, plasmacytoid dendritic cell, T cell CD4 memory, T cell CD4 Th1, T cell CD4 Th2, T cell CD8 naïve | B cell memory, B cell naïve, B cell plasma, macrophage, monocyte, neutrophil, NK cell, T cell CD4 (non−regulatory), T cell CD8, T cell CD8 central memory, T cell gamma delta, T cell regulatory (Tregs) |
|  |  |  |  |
|  |  |  |  |
|  |  |  |  |
|  |  |  |  |
|  |  |  |  |
|  |  |  |  |
|  |  |  |  |
| **Results of intersection** | B cell, endothelial cell, eosinophil, mast cell, T cell CD4 memory resting | T cell CD4 Th2，macrophage M0, T cell CD4 memory activated | - |
|  |  |  |  |
