# Supplementary material for: A tumor mutational burden-derived immune computational framework selects sensitive immunotherapy/chemotherapy for lung adenocarcinoma populations with different prognoses
Source: Front Oncol. 2023 Jun 30;13:1104137. doi: 10.3389/fonc.2023.1104137 (PMC10349266; doi:10.3389/fonc.2023.1104137)
Supplement: Supplementary file 7 [file Table_5.docx]

**Table S5.** The 57 genes upregulated in the high TILPI group.

| **Gene** | **LowMean** | **HighMean** | **Log2(FC)** | **P value** |
| --- | --- | --- | --- | --- |
| **CREB3L3** | 10.7700 | 81.0589 | 2.9120 | 0.0107 |
| **H1-5** | 34.1560 | 164.0057 | 2.2635 | 0.0152 |
| **F2** | 48.2600 | 202.4392 | 2.0686 | 0.0001 |
| **H3C7** | 7.8880 | 31.7072 | 2.0071 | 0.0031 |
| **AC008163.1** | 29.0220 | 102.7167 | 1.8235 | 0.0085 |
| **AC079163.2** | 4.1020 | 13.6939 | 1.7391 | 0.0201 |
| **AC079949.1** | 10.5580 | 34.9848 | 1.7284 | 0.0080 |
| **DSG4** | 4.0000 | 13.1293 | 1.7147 | 0.0243 |
| **H3C11** | 5.2460 | 16.9563 | 1.6925 | 0.0243 |
| **H3C2** | 20.8900 | 63.2966 | 1.5993 | 0.0098 |
| **LINC01913** | 9.9120 | 29.5361 | 1.5752 | 0.0121 |
| **TFF1** | 1290.0900 | 3796.9449 | 1.5574 | 0.0363 |
| **TUBBP6** | 1.4300 | 4.2034 | 1.5555 | 0.0254 |
| **PRR20G** | 12.5560 | 36.7548 | 1.5496 | 0.0136 |
| **H2AC16** | 6.8700 | 19.9601 | 1.5387 | 0.0322 |
| **INHBE** | 17.1540 | 48.9316 | 1.5122 | 0.0226 |
| **PTPRN** | 182.7080 | 515.3232 | 1.4959 | 0.0014 |
| **LINC01234** | 37.6760 | 105.9468 | 1.4916 | 0.0005 |
| **AC091173.1** | 3.3580 | 9.3479 | 1.4770 | 0.0017 |
| **AC089983.1** | 14.7500 | 39.5932 | 1.4245 | 0.0001 |
| **H3C12** | 8.9860 | 23.6996 | 1.3991 | 0.0129 |
| **KYNU** | 1285.0020 | 3308.7871 | 1.3645 | 0.0007 |
| **TARID** | 25.8100 | 64.7357 | 1.3266 | 0.0178 |
| **ISM2** | 9.9160 | 24.5817 | 1.3098 | 0.0154 |
| **GFY** | 15.1780 | 37.2510 | 1.2953 | 0.0109 |
| **UGT2B7** | 39.4180 | 95.2015 | 1.2721 | 0.0143 |
| **AC016769.1** | 2.9020 | 6.9506 | 1.2601 | 0.0005 |
| **NDP** | 37.0100 | 88.5057 | 1.2579 | 0.0140 |
| **H2BC14** | 5.4120 | 12.9125 | 1.2545 | 0.0079 |
| **TRIM15** | 52.4820 | 125.1388 | 1.2536 | 0.0005 |
| **LINC00601** | 2.5280 | 5.9829 | 1.2428 | 0.0059 |
| **AC079949.5** | 6.3020 | 14.8783 | 1.2393 | 0.0001 |
| **BEST3** | 5.1380 | 12.0019 | 1.2240 | 0.0012 |
| **AC093894.2** | 2.6940 | 6.2548 | 1.2152 | 0.0013 |
| **CYP24A1** | 3357.1320 | 7717.5114 | 1.2009 | 0.0093 |
| **MAGEA1** | 100.0580 | 229.0095 | 1.1946 | 0.0026 |
| **AC091133.5** | 2.2200 | 5.0646 | 1.1899 | 0.0092 |
| **MIR9-1HG** | 138.2700 | 314.9848 | 1.1878 | 0.0018 |
| **H1-3** | 55.1940 | 125.0399 | 1.1798 | 0.0016 |
| **GAL** | 103.9320 | 233.8631 | 1.1700 | 0.0120 |
| **IGF2BP1** | 296.1940 | 665.8118 | 1.1686 | 0.0023 |
| **MUC13** | 2079.0180 | 4657.9144 | 1.1638 | 0.0032 |
| **PSMD10P2** | 12.9340 | 28.8156 | 1.1557 | 0.0082 |
| **GIP** | 3.5460 | 7.8194 | 1.1409 | 0.0001 |
| **TRPA1** | 29.7760 | 65.0285 | 1.1269 | 0.0006 |
| **AJ003147.2** | 2.7140 | 5.8878 | 1.1173 | 0.0012 |
| **TRIM40** | 5.2960 | 11.4183 | 1.1084 | 0.0105 |
| **NFE4** | 63.9280 | 137.2281 | 1.1021 | 0.0053 |
| **PRL** | 4.1540 | 8.7548 | 1.0756 | 0.0497 |
| **AC020928.1** | 10.9780 | 22.7985 | 1.0543 | 0.0024 |
| **TREML3P** | 25.2820 | 52.4011 | 1.0515 | 0.0088 |
| **AL731533.3** | 9.3240 | 19.2624 | 1.0468 | 0.0054 |
| **AC079949.2** | 50.8840 | 104.4106 | 1.0370 | 0.0002 |
| **TENM3-AS1** | 12.8820 | 26.1711 | 1.0226 | 0.0436 |
| **ABCC2** | 819.0020 | 1659.6179 | 1.0189 | 0.0022 |
| **KLRC2** | 13.8360 | 27.9202 | 1.0129 | 0.0231 |
| **RAB3B** | 320.1500 | 643.6122 | 1.0074 | 0.0001 |
