# Supplementary material for: A tumor mutational burden-derived immune computational framework selects sensitive immunotherapy/chemotherapy for lung adenocarcinoma populations with different prognoses
Source: Front Oncol. 2023 Jun 30;13:1104137. doi: 10.3389/fonc.2023.1104137 (PMC10349266; doi:10.3389/fonc.2023.1104137)
Supplement: Supplementary file 8 [file Table_6.docx]

**Table S6.** Screening of 7 drugs candidate related genes based on CellMiner database.

| **Gene** | **Drug** | **Correlation** | **P value** |
| --- | --- | --- | --- |
| RAB3B | Vinorelbine | -0.41122 | 0.001099 |
| RAB3B | Paclitaxel | -0.38352 | 0.002488 |
| UGT2B7 | Thapsigargin | -0.38271 | 0.002545 |
| RAB3B | Thapsigargin | -0.37785 | 0.002916 |
| CYP24A1 | Vinorelbine | -0.37442 | 0.003206 |
| UGT2B7 | Paclitaxel | -0.3643 | 0.004216 |
| UGT2B7 | Vinorelbine | -0.34798 | 0.00644 |
| TRIM15 | Gemcitabine | -0.33238 | 0.009469 |
| CREB3L3 | Palbociclib | 0.329559 | 0.010131 |
| IGF2BP1 | Pyrimethamine | 0.322151 | 0.012067 |
| GAL | Palbociclib | 0.312605 | 0.015025 |
| RAB3B | Gemcitabine | 0.308282 | 0.016557 |
| BEST3 | Palbociclib | 0.30773 | 0.016762 |
| MUC13 | Gemcitabine | -0.29971 | 0.019992 |
| ABCC2 | Pyrimethamine | 0.299288 | 0.020177 |
| TFF1 | Palbociclib | 0.278796 | 0.030999 |
| DSG4 | Palbociclib | 0.277757 | 0.031657 |
| GIP | Pyrimethamine | 0.276133 | 0.032709 |
| BEST3 | Vinorelbine | -0.26064 | 0.044288 |
